# Tris(*N*-{bis­[meth­yl(phen­yl)amino]­phosphor­yl}benzene­sulfonamidato-κ^2^ *O*,*O*′)(1,10-phenanthroline-κ^2^ *N*,*N*′)lanthanum(III)

**DOI:** 10.1107/S2056989017008970

**Published:** 2017-06-27

**Authors:** Angelina Yu. Prytula-Kurkunova, Victor A. Trush, Viktoriya V. Dyakonenko, Tetyana Yu. Sliva, Vladimir M. Amirkhanov

**Affiliations:** a National Taras Shevchenko University of Kyiv, Department of Chemistry, 12 Lva Tolstogo str., 01033 Kyiv, Ukraine; b STC "Institute for Single Crystals", 60 Nauki ave., Kharkiv 61072, Ukraine

**Keywords:** crystal structure, phospho­rylated sulfonyl­amide, sulfonyl­amido­phosphate, π–π stacking, eight-coordinated lanthanide complex

## Abstract

The asymmetric unit of [La(C_20_H_21_N_3_O_3_PS)_3_(C_12_H_8_N_2_)] is created by one La^III^ ion, three deprotonated *N*-{bis­[meth­yl(phen­yl)amino]­phosphor­yl}benzene­sulfonamidate (*L* ^−^) ligands and one 1,10-phenanthroline (Phen) mol­ecule. Each La^III^ ion is eight-coordinated (6O+2N) by three phosphoryl O atoms, three sulfonyl O atoms of three *L* ^−^ ligands and two N atoms of the chelating Phen ligand, leading to the formation of six- and five-membered metallacycles, respectively. The lanthanum coordination polyhedron has a bicapped trigonal–prismatic geometry. ‘Sandwich-like’ intra­molecular π–π stacking inter­actions are observed between the 1,10-phenanthroline ligand and two benzene rings of two different *L* ^−^ ligands. The phenyl rings of *L* ^−^ that are not involved in the stacking inter­actions show minor positional disorder. Mol­ecules form layers parallel to the (010) plane due to weak C—H⋯O inter­molecular hydrogen bonds. Unidentified highly disordered solvate mol­ecules that occupy *ca* 400 Å^3^ large voids have been omitted from the refinement model.

## Abstract 2

A lanthanum(III) complex with formula La*L*_3_Phen (where *L* ^−^ is the sulfonyl­amido­phosphate (SAPh)-type ligand *N*-{bis­[meth­yl(phen­yl)amino]­phosphor­yl}benzene­sulfonamidate, C_6_H_5_SO_2_NHPO[N(CH_3_)C_6_H_5_]_2_ has been synthesized and its crystal structure determined.

## Chemical context   

β-Diketone derivatives have been the topic of investigations in many different branches of the chemical science, such as organic, coordination, bio- and theoretical chemistry. Of special inter­est have been carbacyl­amido­phosphates (CAPh), containing the functional fragment C(O)NHP(O), because of their properties as extractants (Morgalyuk *et al.*, 2005 ▸; Safiulina *et al.*, 2015 ▸), urease inhibitors (Jaroslav & Swerdloff, 1985 ▸), enzyme inhibitors (Grimes *et al.*, 2008 ▸; Adams *et al.*, 2002 ▸), their anti­bacterial properties (Oroujzadeh *et al.*, 2017 ▸) and anti­cancer activity (Kovalchyk *et al.*, 1991 ▸; Amirkhanov *et al.*, 1995 ▸). The presence of the phosphoryl group gives them a high affinity towards highly charged metal ions, and these types of compounds are used in the coordination chemistry of lanthanides and actinides (Litsis *et al.*, 2010 ▸, 2017 ▸; Kariaka *et al.*, 2013 ▸). Many efforts have been devoted to the synthesis of another type of structural analogs of β-diketones – sulfonyl­amido­phosphates (SAPh) with the structural fragment S(O)_2_NHP(O). These types of compounds were first synthesized by Kirsanov (Kirsanov & Shevchenko, 1954 ▸) and some have since been used as bactericidal agents in medicine and toxicology (Xu & Angell, 2000 ▸), while others have found use as pesticides (Kishino & Saito, 1979 ▸). In addition, these compounds are potentially bidentate *O*,*O*-donor chelating ligands for metal ions, similar to other deprotonated phospho­rylic ligand derivatives (Znovjyak *et al.*, 2015 ▸; Amirkhanov *et al.*, 2014 ▸; Litsis *et al.*, 2016 ▸; Shatrava *et al.*, 2016*a* ▸). For details of the coordination chemistry of phospho­rylic ligands in mol­ecular form, see Gholivand *et al.* (2012 ▸, 2014 ▸), Yizhak *et al.* (2013 ▸) and Shatrava *et al.* (2016*b* ▸).

Recently, we reported the preparation and study of the coordination properties of several representatives of sulfonyl­amido­phosphates: meth­yl(phenyl­sulfon­yl)amido­phosphate [PhSO_2_NHP(O)(OMe)_2_] (Moroz *et al.*, 2007 ▸) and particularly the photophysical properties of a series of NIR-emitting lanthanide complexes (Kulesza *et al.*, 2010 ▸). It was shown that the solid-state decay time for the ytterbium complex is one of the longest of all known Yb^III^ complexes with organic ligands. It is expected that depending on the nature of substituents attached to the phospho­rus and sulfur atoms, these organic compounds and their complexes might demonstrate unique specific physicochemical properties. Optical studies of the etheric type SAPh ligands dimeth­yl(4-methyl­phenyl­sulfon­yl)amido­phosphate [(Me)PhSO_2_NHP(O)(OMe)_2_] and dimethyl 2-naphthyl­sulfonyl­amido­phosphate [(C_10_H_7_)SO_2_NHP(O)(OMe)_2_] indicate that the ligand first excited singlet state plays a dominant role in intra­molecular energy transfer processes in these *Ln* complexes (Kasprzycka *et al.*, 2016 ▸).

Knowledge of the crystal structure is an essential part of understanding the luminescent properties of these types of lanthanide complexes. In this paper we would therefore like to report the mol­ecular and crystal structure of a lanthanum coordination compound based on the amidic type SAPh ligand *N*-(meth­yl(phenyl­amino)­phosphor­yl)benzene­sulfon­amide (**HL**) [PhSO_2_NHP(O)(N(Me)Ph)_2_] with the general formula **La(L)_3_Phen**.
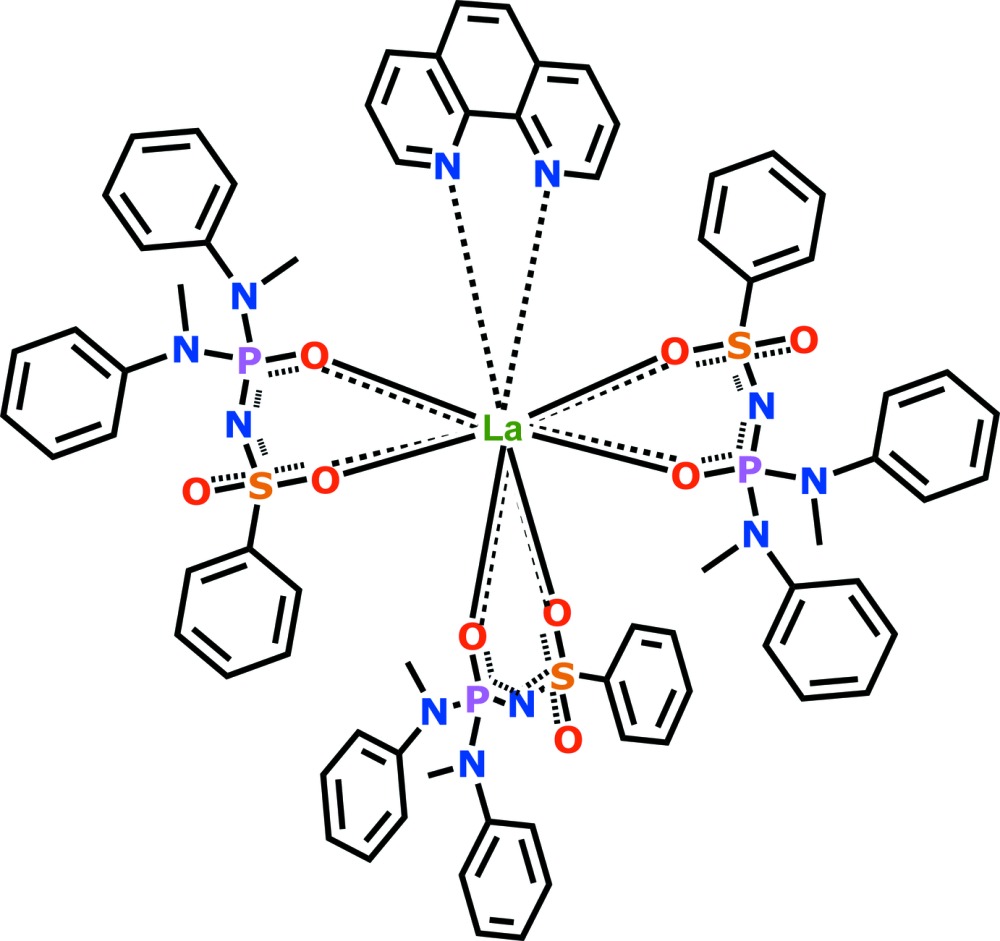


## Structural commentary   

**Figure 1:**
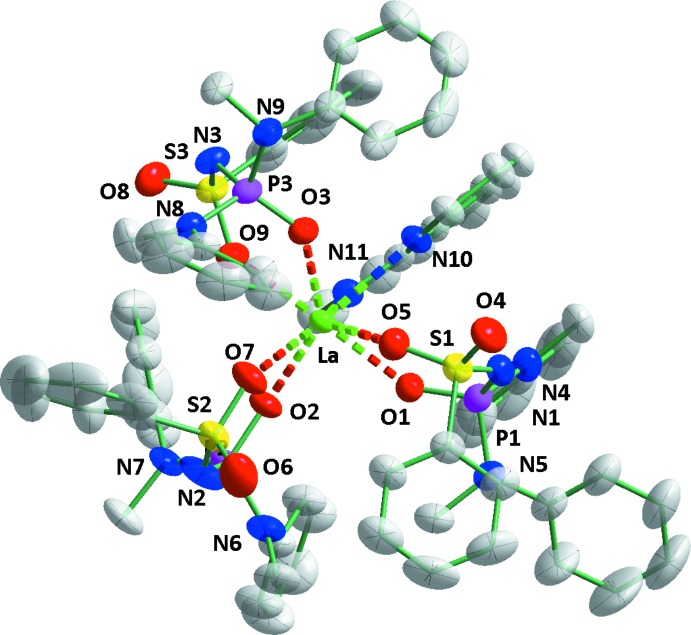
Structural representation of **LaL_3_Phen** with partial atom-numbering scheme. Displacement ellipsoid are drawn at the 50% probability level and H atoms have been omitted for clarity.

The title compound **La(L)_3_Phen** crystallizes with one mol­ecule in the asymmetric unit (Fig. 1 ▸). The coordination environment of the La atom consists of two nitro­gen atoms of 1,10-phenanthroline and six oxygen atoms from the three acido-SAPh ligands.

The La—O(S) bond lengths [2.516 (2)–2.541 (2) Å] are all longer than those of their La—O(P) counterparts [2.424 (2)–2.463 (2) Å], with mean values of 2.435 and 2.456 Å, respectively. The mean average of all La—O bond lengths is 2.476 Å. The La—N distances are with 2.699 (3) and 2.700 (3) Å (mean value 2.693 Å) shorter than those previously obtained for a 1,10-phenanthrolinate lanthanum (III) complex with hexa­fluoro­acetyl­acetonate (2.747–2.782 Å; Rogachev *et al.*, 2005 ▸) and longer than La—N bonds in a carbacyl­amido­phosphate ligand complex (2.601–2.635 Å; Litsis *et al.*, 2015 ▸; Sokolnicki *et al.*, 1999 ▸).

The SAPh ligands coordinate to the lanthanide atom in the acido form in a bidentate manner with formation of six-membered metallocycles with partial delocalization of π-electron density. The values of the S—O and P—O bonds are at 1.462 (3)–1.474 (2) Å and 1.491 (2)–1.494 (2) Å in their expected ranges. The mean values are 1.468 and 1.492 Å, respectively. The corresponding bond lengths in the related neutral ligands are around 1.42 Å (Moroz *et al.*, 2012 ▸) and 1.48 Å (Znovjyak *et al.*, 2009 ▸). The S—O bonds of the SAPh ligands of the non-coordinating oxygen atom are systematically shorter [1.432 (3)–1.437 (3) Å], indicating more S=O double-bond character than for the coordinating O atoms.

The six-membered metallocyclic rings with the chelate (O)PNS(O) fragments are all non-planar. The La1–O1–P1–N1–S1–O1 (*A*) and La1–O3–P3–N3–S3–O9 (*B*) rings both adopt twist-boat conformations (puckering parameters are: *S* = 0.61, ψ = 22.11°, θ = 79.68° for *A* and *S* = 0.75, ψ = 24.44°, θ = 87.28° for *B*, respectively (Zefirov *et al.*, 1990 ▸)). The deviations of the N1 and O1 atoms from the mean plane through the remaining atoms of *A* (r.m.s.deviation = 0.06 Å) are 0.78 and 0.41 Å, respectively. The deviations of the La1 and O3 atoms from the mean plane through the remaining atoms of *B* (r.m.s.deviation = 0.06 Å) are 0.9 and 0.88 Å, respectively. The La1–O2–P2–N2–S2–O7 (*C*) ring adopts a flattened half-chair conformation (puckering parameters are: *S* = 0.71, ψ = 16.51°, θ = 20.43°). The deviation of the La1 atom from the mean plane carried through the remaining atoms of ring *C* (r.m.s.deviation 0.02 Å) is 0.36  Å.

**Figure 2:**
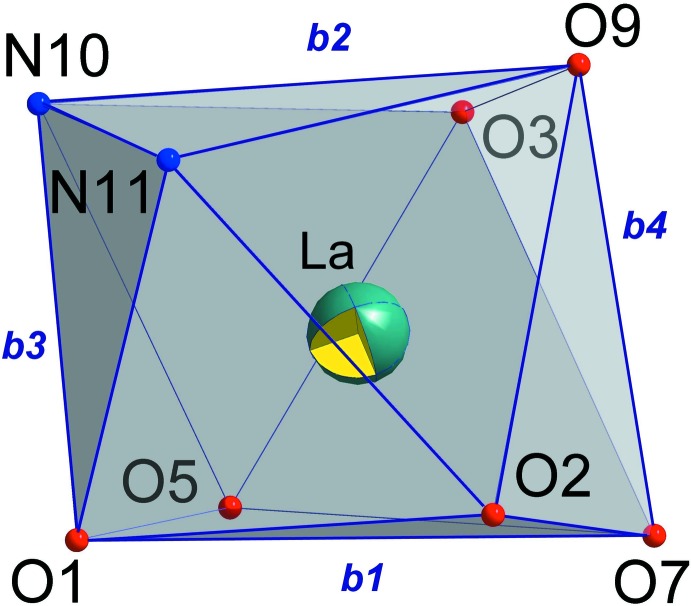
The coordination polyhedron around the central La^III^ atom in **LaL_3_Phen** with *b* parameters indicated.

The *δ*-criterions were used to characterize the lanthanum ion eight-apical coordination polyhedron (Porai-Koshits & Aslanov, 1972 ▸). The set of the angles *δ* between pairs of the faces inter­secting along the type *b* edges (shown in Fig. 2 ▸) allows us to assign a distorted bicapped trigonal–prismatic environment (*δ*_1_ = 9.48°, *δ*_2_ = 18.48°, *δ*_3_ = 43.57°, *δ*_4_ = 44.89°, *φ*_1_ = 12.47°, *φ*_2_ = 16.05°) similar to that of the Tb(Pip)_3_(Phen) mixed-ligand complex with 2,2,2-tri­chloro-*N*-(dipiperidin-1-yl-phosphor­yl)acetamide, HPip (Litsis *et al.*, 2015 ▸).

Intra­molecular ‘sandwich-like’ π–π-stacking inter­actions are observed between the 1.10-phenanthroline fragments and two phenyl rings of the two SAPh ligands of the title mol­ecule. The central ring C66(π)⋯C69(π) of the 1,10-phenanthroline mol­ecule inter­acts with the C41(π)⋯C46(π) phenyl ring at the sulfonyl group from another ligand [inter­planar angle 2.8 (1)°, inter­centroid distance 3.646 (2) Å, inter­planar separation 3.38–3.41 Å, plane shift 1.29–1.36 Å], and with the C8(π)⋯C13(π) ring at the phosphoryl group from the other ligand [inter­planar angle 2.5 (2)°, inter­centroid distance 3.879 (3) Å, inter­planar separation 3.49–3.52 Å, plane shift 1.62–1.69 Å]. A similar intra­molecular organization was described previously for related compounds (Beloso *et al.*, 2003 ▸).

## Supra­molecular features   

**Figure 3:**
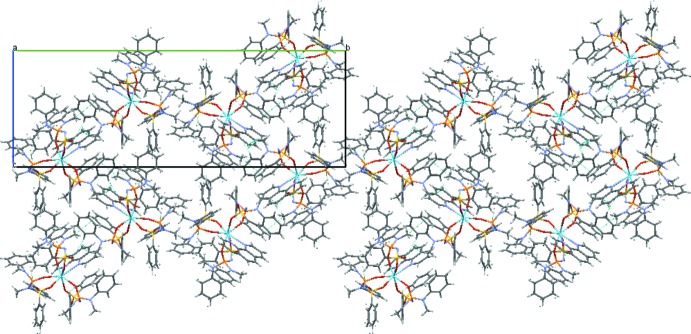
The crystal packing of **LaL_3_Phen**. The view is along the crystallographic *a* axis.

In the crystal phase, the **La(L)_3_Phen** mol­ecules are linked by weak C—H⋯O hydrogen bonds (Table 1 ▸), forming double layers parallel to the (010) plane (Fig. 3 ▸). There are solvent-accessible voids with a total volume of 380 Å^3^. The content of the voids is not resolved in difference-density maps, with the largest residual electron density peak being only 0.66 electrons per Å^3^. A SQUEEZE (Spek, 2015 ▸) analysis indicated an overall electron count matching approximately three mol­ecules of the solvent (2-propanol) per unit cell, but did not improve *R* values or other quality indicators (see *Refinement* section).

## Database survey   

A search of the Cambridge Structural Database (CSD, Version 5.38, update February 2017; Groom *et al.*, 2016 ▸) for SAPh ligand analogues with derivatives of the *N*-(bis­(di­amino)­phosphor­yl)sulfonamide fragments yielded five hits, with only one metal complex structure with a neodymium metal atom among them (Shatrava *et al.*, 2010 ▸). In this mol­ecule, the neodymium atom is also octa­coordinated, with a highly symmetrical NdO_8_ polyhedron and no coordinating N atoms.

A search for phenanthrolinate REE complexes with other SAPh-type ligands returned one entry for tris­(dimethyl (phenyl­sulfon­yl)phospho­ramidato-*O*,*O*′)-(1,10-phenanthroline-*N*,*N*′)-erbium(III) (SAPHICP; Gawryszewska *et al.*, 2011 ▸).

A search for octa­coordinated La complexes with an LaN_2_O_6_ environment yielded 20 hits, with average La—O and La—N bond lengths of 2.476 and 2.693 Å, respectively. 11 complex structures with different lanthanoid metals (*Ln*) containing *Ln*–O–P–N–S–O metallocycles were found in the database, all with octa­coordinated metal atoms. Most of those metallacyclic rings are non-planar with mean deviations of the O and N atoms of 0.329 and 0.434 Å, respectively.

## Synthesis and crystallization   

^1^H and ^31^P NMR spectra in DMSO-*d*_6_ solutions were recorded on a Varian 400 NMR spectrometer at room temperature. ^1^H chemical shifts were determined relative to the inter­nal standard TMS whereas ^31^P chemical shifts were determined relative to 85% H_3_PO_4_ as an external standard. Infrared (FTIR) spectra were recorded on a Perkin–Elmer Spectrum BX spectrometer using KBr pellets. The resolution of the FTIR spectra is 1 cm^−1^.

**Sulfonyl­amido­phosphate ligand** *N*-(meth­yl(phenyl­amino)­phosphor­yl)benzene­sulfonamide (**HL**) was synthesized *via* a three-step procedure based on the Kirsanov reaction (Kirsanov & Shevchenko, 1954 ▸). ^1^H NMR (400 MHz, DMSO-*d*_6_, 293 K) *δ* 2.95 (*d*, *J* = 7.6, 6H, CH_3_), 7.05 (*t*, *J* = 5.6, 2H, *γ*-CH_phenyl­amino_), 7.14 (*d*, *J* = 6.4, 4H, *α*-CH_phenyl­amino_), 7.21 (*t*, *J* = 6.2, 4H, β-CH_phenyl­amino_), 7.56 (*t*, *J* = 6.2, 2H, β-CH), 7.65 (*t*, *J* = 6.2, 1H, *γ*-CH), 7.91 (*d*, *J* = 6.0, 2H, *α*-CH).

IR (KBr pellet, cm^−1^): 3062 [*m*, ν (C—H_aliph_)], 2948 [*m*, ν (C—H_arom_)], 2780 [*m*, ν(N—H)], 2705 [*m*, ν(C—H_arom_)], 2655 [w, ν(C—H_arom_)], 1594 [*s*, ν(S=N)], 1495 (*s*), 1446 (*m*), 1400 (*m*), 1330 [*s*, ν(S=O_2_)], 1279 (m), 1220 [*ws*, ν(P=O)], 1168 [*s*, ρ(CH_3_)], 1084 (*m*), 1069 (*m*), 1028 (*m*), 920 [*ws*, ν(P—N)], 887 (*ws*), 765 (*s*), 758 (*s*), 723 (*m*), 696 (*s*), 685 (*s*), 602 (*m*), 573 (*m*), 558 (*m*), 551 (*m*), 542 (*m*), 508 (*s*), 490 (*m*), 442 (*w*).

The sodium salt (**NaL**) was prepared by the reaction between equimolar amounts of sodium methano­late (0.069 g, 3 mmol of Na was dissolved in 20 ml of methanol) and HL (1.39 g, 3 mmol) in an methanol medium (20 ml). The mixture was heated with magnetic stirring at 337 K for 10 min. The resulting solution was evaporated and the fine crystalline powder was isolated (yield 83%) and washed with 2-propanol. Dry product **NaL** was used for the preparation of the complexes. ^1^H NMR (400 MHz, DMSO-*d*_6_, 290 K) *δ* 3.46 (*s*, 3H, CH_3_), 3.48 (*s*, 3H, CH_3_), 7.26 (*t*, *J* = 7.2, 2H, *γ*-CH_phenyl­amino_), 7.53 (*t*, *J* = 8, 4H, β-CH_phenyl­amino_), 7.6 (*d*, *J* = 8.4, 4H, *α*-CH_phenyl­amino_), 7.77 (*m*, 5H, CH). ^31^P NMR (400 MHz, DMSO-*d*_6_, 290 K) *δ* 54.01.

IR (KBr pellet, cm^−1^): 3068 [*m*, ν (C—H_aliph_)], 2944 [*m*, ν(C—H_arom._)], 2704 [*m*, ν(C—H_arom_)], 2660 [*w*, ν(C—H_arom_)], 1581 [*s*, ν(S=N)], 1490 (*s*), 1410 [*m*, ν(C=C)], 1263 [*s*, ν(S=O_2_)], 1271 (*m*), 1173 [*ws*, ν(P=O)], 1165 [*s*, ρ(CH_3_)], 1080 (*m*), 1031 (*m*), 891 [*ws*, ν(P—N], 870 (*ws*), 761 (*s*), 747 (*s*), 720 (*m*), 695 (*s*), 680 (*s*), 573 (*m*), 551 (*m*), 540 (*m*), 503 (*s*), 485 (*m*), 432 (*w*).

**Preparation of La(L)_3_Phen.** **NaL** (0.728 g, 1.5 mmol) was dissolved in 7 ml of 2-propanol and was added to a solution of 1,10-phenanthroline monohydrate (0.0991 g, 0.5 mmol) in 2 ml of 2-propanol. Then the mixture was heated to 340 K and poured into a solution of La(NO_3_)_3_·6H_2_O (0.216 g, 0.5 mmol) in 5 ml of 2-propanol heated to 340 K. After 10 minutes, the resulting mixture was filtered from sodium nitrate and the filtrate was left in a desiccator above CaCl_2_ at room temperature. Similar compounds were obtained for Ln^3+^ = Pr, Nd, Eu, Ho, Tb and Lu.

Crystals of the complexes formed after 1–2 days, were filtered and washed with cooled 2-propanol and dried in air (yield 82-86%). The complexes, as prepared, are soluble in non-polar aprotic solvents, and are less soluble in acetone and alcohols. Crystalline powder of **La(L)_3_Phen** was recrystallized from a 2-propanol/methanol mixture (5:1, *v*/*v*) to give colourless prisms (0.65 g, 0.5 mmol, 84%). ^1^H NMR (400 MHz, DMSO-*d*_6_, 293 K) *δ* 2.87 (*s*, 9H, CH_3_), 2.9 (*s*, 9H, CH_3_), 7.32 (*t*, *J* = 7.2, 6H, *γ*-CH_phenyl­amino_), 7.57 (*t*, *J* = 8, 12H, β-CH_phenyl­amino_), 7.62 (*d*, *J* = 8.4, 12H, *α*-CH_phenyl­amino_), 7.74 (*m*, 15H, CH),7.69 (*m*, 2H, Phen), 7.89 (*m*, 2H, Phen), 8.41 (*d*, 2H, Phen), 9.12 (*d*, 2H, Phen). ^31^P NMR (400 MHz, DMSO-*d*_6_, 290 K) *δ* 45.1.

IR (KBr pellet, cm^−1^): 3067 [*m*, ν (C—H_aliph_)], 2943 [*m*, ν (C—H_arom_)], 2704 [*m*, ν(C-H_arom_)], 2660 [*w*, ν(C—H_arom_)], 1564 [*m*, ν(C=N], 1572 [*s*, ν(S=N)], 1490 (*s*), 1413 [*m*, ν(C=C)], 1252 [*s*, ν(S=O_2_)], 1243 [*m*, ν(C—N] + ν(C—C)], 1270 (*m*), 1164 [*ws*, ν(P=O)], 1165 [*s*, ρ(CH_3_)], 1082 (*m*), 1030 (*m*), 990 [*m*, δ(CCN_amine_)], 892 [*ws*, ν(P—N], 874 (*ws*), 763 (*s*), 747 (*s*), 720 (*m*), 678 (*s*), 682 (*s*), 564 (*m*), 547 (*m*), 534 (*m*), 502 (*s*), 485 (*m*), 427 (*w*).

## Refinement   

Crystal data, data collection and structure refinement details are summarized in Table 2 ▸. All H atoms were positioned geometrically and refined using a riding model, with C—H = 0.93–0.96 Å and *U*_iso_(H) = *xU*_eq_(C), where *x* = 1.5 for methyl H and 1.2 for all other H atoms. A rotating-group model was applied for the methyl groups.

Phenyl ring C1–C6 was refined as disordered over two positions *A* and *B* with refined occupancies of 0.50 (3) for both disorder components. The phenyl rings C15–C20, C21–C26 were refined as disordered over two positions with refined occupancies of 0.555 (17) and 0.445 (17), respectively. The bond lenghts C21*A*—C22*A*, C22*A*—C23*A*, C23*A*—C24*A*, C24*A*—C25*A*, C25*A*—C26*A*, C26*A*—C21*A*, C21—C22, C22—C23, C23—C24, C24—C25, C25—C26 and C26—C21 were restrained to have a value of 1.38 (1) Å (using a DFIX restraint). The ring carbon atoms C21*A*, C26*A*, C25*A*, C24*A*, C23*A*, C22*A* as well as C21, C22, C23, C24, C25, C26 were restrained to have planar geometries (within 0.01 Å, using a FLAT restraint). Anisotropic parameters of all C atoms of disordered rings were restrained to have approximately similar values to within 0.01 Å^2^ (using a SIMU restraint).

During the refinement, several small isolated electron-density peaks were located in solvent-accessible voids that were believed to be solvent mol­ecules. The largest residual electron peak accounted to 0.66 e Å^3^. Satisfactory results (*R*_1_ = 5.01%) were obtained modeling disordered C and O atoms, but very large displacement parameters for them were observed. The SQUEEZE procedure (Spek, 2015 ▸) implemented in *PLATON* indicated two solvent cavities each of volume 380 A^3^, each containing approximately 52 electrons, which corresponds to approximately three mol­ecules of the solvent (2-propanol) per cell. However, the difference in *R*_1_ values for the structures with and without the SQUEEZE procedure implemented was rather small (0.5%). In the final refinement, the isolated peaks in the solvent-accessible voids were ignored.

## Supplementary Material

Crystal structure: contains datablock(s) I, New_Global_Publ_Block. DOI: 10.1107/S2056989017008970/zl2701sup1.cif

Supporting information file. DOI: 10.1107/S2056989017008970/zl2701Isup4.cdx

Structure factors: contains datablock(s) I. DOI: 10.1107/S2056989017008970/zl2701Isup6.hkl

hkl-file. DOI: 10.1107/S2056989017008970/zl2701sup5.txt

CCDC reference: 1556367

Additional supporting information:  crystallographic information; 3D view; checkCIF report

## Figures and Tables

**Table 1:** Hydrogen-bond geometry (Å, °)

*D*—H⋯*A*	*D*—H	H⋯*A*	*D*⋯*A*	*D*—H⋯*A*
C70—H70⋯O4^i^	0.93	2.67	3.444 (6)	139
C56—H56⋯O4^ii^	0.93	2.71	3.448 (5)	136

Symmetry codes: (i) 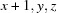; (ii) 
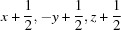
.

**Table 2:** Experimental details

Crystal data	
Chemical formula	[La(C_20_H_21_N_3_O_3_PS)_3_(C_12_H_8_N_2_)]
*M*_r_	1562.39
Crystal system, space group	Monoclinic, *P*2_1_/*n*
Temperature (K)	293
*a*, *b*, *c* (Å)	12.2213 (2), 42.2455 (7), 15.5956 (3)
β (°)	108.222 (2)
*V* (Å^3^)	7648.1 (2)
*Z*	4
Radiation type	Mo *K*α
μ (mm^−1^)	0.76
Crystal size (mm)	0.3 × 0.2 × 0.1

Data collection	
Diffractometer	Agilent Xcalibur Sapphire3
Absorption correction	Multi-scan (*CrysAlis PRO*; Agilent, 2016 ▸)
*T*_min_, *T*_max_	0.963, 1.000
No. of measured, independent and observed [*I* > 2σ(*I*)] reflections	43241, 16526, 13120
*R*_int_	0.027
(sin θ/λ)_max_ (Å^−1^)	0.654

Refinement	
*R*[*F* ^2^ > 2σ(*F* ^2^)], *wR*(*F* ^2^), *S*	0.047, 0.119, 1.08
No. of reflections	16526
No. of parameters	1015
No. of restraints	576
H-atom treatment	H-atom parameters constrained
Δρ_max_, Δρ_min_ (e Å^−3^)	0.66, −0.48

Computer programs: *CrysAlis PRO* (Agilent, 2016 ▸), *SHELXT* (Sheldrick, 2015*a* ▸), *SHELXL2016* (Sheldrick, 2015*b* ▸) and *OLEX2* (Dolomanov *et al.*, 2009 ▸).

## supplementary crystallographic information

### Crystal data

**Table:**

[La(C_20_H_21_N_3_O_3_PS)_3_(C_12_H_8_N_2_)]	*F*(000) = 3208
*M**_r_* = 1562.39	*D*_x_ = 1.357 Mg m^−^^3^
Monoclinic, *P*2_1_/*n*	Mo *K*α radiation, λ = 0.71073 Å
*a* = 12.2213 (2) Å	Cell parameters from 14527 reflections
*b* = 42.2455 (7) Å	θ = 2.9–27.6°
*c* = 15.5956 (3) Å	µ = 0.76 mm^−^^1^
β = 108.222 (2)°	*T* = 293 K
*V* = 7648.1 (2) Å^3^	Block, colourless
*Z* = 4	0.3 × 0.2 × 0.1 mm

### Data collection

**Table:**

Agilent Xcalibur Sapphire3 diffractometer	16526 independent reflections
Radiation source: Enhance (Mo) X-ray Source	13120 reflections with *I* > 2σ(*I*)
Graphite monochromator	*R*_int_ = 0.027
Detector resolution: 16.1827 pixels mm^-1^	θ_max_ = 27.7°, θ_min_ = 2.9°
ω scans	*h* = −15→15
Absorption correction: multi-scan (CrysAlis PRO; Agilent, 2016)	*k* = −49→55
*T*_min_ = 0.963, *T*_max_ = 1.000	*l* = −16→19
43241 measured reflections	

### Refinement

**Table:**

Refinement on *F*^2^	576 restraints
Least-squares matrix: full	Hydrogen site location: inferred from neighbouring sites
*R*[*F*^2^ > 2σ(*F*^2^)] = 0.047	H-atom parameters constrained
*wR*(*F*^2^) = 0.119	*w* = 1/[σ^2^(*F*_o_^2^) + (0.0547*P*)^2^ + 4.2326*P*] where *P* = (*F*_o_^2^ + 2*F*_c_^2^)/3
*S* = 1.08	(Δ/σ)_max_ = 0.004
16526 reflections	Δρ_max_ = 0.66 e Å^−^^3^
1015 parameters	Δρ_min_ = −0.48 e Å^−^^3^

### Special details

**Table:**

Geometry. All esds (except the esd in the dihedral angle between two l.s. planes) are estimated using the full covariance matrix. The cell esds are taken into account individually in the estimation of esds in distances, angles and torsion angles; correlations between esds in cell parameters are only used when they are defined by crystal symmetry. An approximate (isotropic) treatment of cell esds is used for estimating esds involving l.s. planes.

### Fractional atomic coordinates and isotropic or equivalent isotropic displacement parameters (Å^2^)

**Table:**

	*x*	*y*	*z*	*U*_iso_*/*U*_eq_	Occ. (<1)
La1	0.09120 (2)	0.35503 (2)	0.43119 (2)	0.03134 (6)	
S1	−0.21614 (8)	0.34454 (2)	0.28067 (7)	0.0450 (2)	
S2	−0.00520 (9)	0.42066 (2)	0.55977 (7)	0.0483 (2)	
S3	0.33083 (7)	0.31661 (2)	0.61269 (6)	0.0430 (2)	
P1	−0.06748 (9)	0.36460 (2)	0.18902 (6)	0.0439 (2)	
P2	0.15801 (9)	0.44041 (2)	0.47608 (7)	0.0480 (2)	
P3	0.11147 (8)	0.30133 (2)	0.61951 (6)	0.0378 (2)	
O1	0.0235 (2)	0.37287 (6)	0.27530 (15)	0.0435 (6)	
O2	0.1744 (2)	0.40764 (5)	0.44636 (17)	0.0432 (6)	
O3	0.05751 (19)	0.31109 (5)	0.52365 (15)	0.0393 (5)	
O4	−0.2988 (2)	0.31980 (6)	0.2749 (2)	0.0610 (8)	
O5	−0.1245 (2)	0.34570 (6)	0.36742 (17)	0.0475 (6)	
O6	−0.1236 (3)	0.43021 (9)	0.5311 (2)	0.0824 (10)	
O7	0.0112 (2)	0.38806 (6)	0.53354 (18)	0.0522 (7)	
O8	0.4363 (2)	0.32486 (7)	0.68112 (19)	0.0567 (7)	
O9	0.27345 (19)	0.34382 (6)	0.55776 (17)	0.0434 (6)	
N1	−0.1691 (3)	0.34263 (8)	0.1991 (2)	0.0484 (8)	
N2	0.0722 (4)	0.44426 (8)	0.5338 (3)	0.0708 (11)	
N3	0.2496 (2)	0.29808 (7)	0.6524 (2)	0.0441 (7)	
N4	−0.0168 (3)	0.34447 (8)	0.1181 (2)	0.0544 (8)	
N5	−0.1155 (3)	0.39885 (8)	0.1385 (2)	0.0544 (8)	
N6	0.1081 (3)	0.46540 (7)	0.3910 (2)	0.0550 (8)	
N7	0.2845 (3)	0.45430 (7)	0.5354 (2)	0.0581 (9)	
N8	0.0813 (3)	0.32513 (7)	0.6930 (2)	0.0453 (7)	
N9	0.0579 (2)	0.26692 (7)	0.63781 (19)	0.0424 (7)	
N10	0.0939 (3)	0.30261 (7)	0.3329 (2)	0.0427 (7)	
N11	0.2631 (2)	0.34687 (7)	0.3593 (2)	0.0405 (7)	
C1	−0.2863 (19)	0.3812 (3)	0.2737 (14)	0.052 (2)	0.50 (3)
C2	−0.3688 (18)	0.3912 (4)	0.1948 (12)	0.061 (2)	0.50 (3)
H2	−0.392524	0.377731	0.145258	0.074*	0.50 (3)
C3	−0.4157 (13)	0.4214 (4)	0.1900 (12)	0.069 (3)	0.50 (3)
H3	−0.470864	0.428082	0.137292	0.083*	0.50 (3)
C4	−0.3802 (13)	0.4415 (3)	0.2641 (14)	0.069 (3)	0.50 (3)
H4	−0.411601	0.461676	0.260914	0.083*	0.50 (3)
C5	−0.2978 (14)	0.4315 (3)	0.3429 (12)	0.068 (3)	0.50 (3)
H5	−0.273998	0.444919	0.392501	0.081*	0.50 (3)
C6	−0.2508 (16)	0.4013 (4)	0.3477 (12)	0.059 (2)	0.50 (3)
H6	−0.195657	0.394568	0.400468	0.070*	0.50 (3)
C7	−0.0699 (4)	0.31454 (12)	0.0784 (3)	0.0752 (14)	
H7A	−0.104419	0.317109	0.014435	0.113*	
H7B	−0.128013	0.308591	0.104901	0.113*	
H7C	−0.012079	0.298327	0.089899	0.113*	
C8	0.0783 (4)	0.35548 (12)	0.0932 (3)	0.0619 (12)	
C9	0.1344 (4)	0.38299 (14)	0.1247 (3)	0.0779 (14)	
H9	0.108845	0.395682	0.163289	0.094*	
C10	0.2290 (5)	0.39237 (18)	0.1001 (4)	0.104 (2)	
H10	0.265300	0.411399	0.121904	0.124*	
C11	0.2698 (6)	0.3743 (2)	0.0446 (5)	0.117 (3)	
H11	0.333153	0.380865	0.028254	0.140*	
C12	0.2173 (6)	0.3471 (2)	0.0142 (4)	0.116 (3)	
H12	0.245607	0.334365	−0.022779	0.139*	
C13	0.1217 (5)	0.33734 (15)	0.0363 (3)	0.0872 (17)	
H13	0.085688	0.318426	0.012907	0.105*	
C14	−0.0865 (5)	0.42850 (10)	0.1906 (3)	0.0755 (14)	
H14A	−0.026621	0.439343	0.174686	0.113*	
H14B	−0.060492	0.423703	0.253986	0.113*	
H14C	−0.153577	0.441758	0.177217	0.113*	
C15	−0.1910 (9)	0.4014 (3)	0.0472 (7)	0.068 (2)	0.555 (17)
C20	−0.2460 (12)	0.3743 (3)	0.0040 (9)	0.083 (2)	0.555 (17)
H20	−0.231449	0.354876	0.033288	0.100*	0.555 (17)
C19	−0.3227 (11)	0.3763 (2)	−0.0830 (9)	0.096 (2)	0.555 (17)
H19	−0.359468	0.358198	−0.111961	0.115*	0.555 (17)
C18	−0.3444 (10)	0.4054 (3)	−0.1268 (6)	0.107 (3)	0.555 (17)
H18	−0.395734	0.406726	−0.185036	0.128*	0.555 (17)
C17	−0.2894 (13)	0.4325 (2)	−0.0836 (6)	0.109 (3)	0.555 (17)
H17	−0.303983	0.451934	−0.112863	0.131*	0.555 (17)
C16	−0.2127 (11)	0.4305 (3)	0.0035 (6)	0.095 (3)	0.555 (17)
H16	−0.175963	0.448613	0.032386	0.114*	0.555 (17)
C21	0.0248 (11)	0.4209 (3)	0.6771 (10)	0.068 (2)	0.566 (10)
C22	0.1270 (12)	0.4307 (3)	0.7356 (7)	0.090 (2)	0.566 (10)
H22	0.184482	0.438013	0.713144	0.108*	0.566 (10)
C23	0.1468 (12)	0.4301 (3)	0.8282 (7)	0.107 (3)	0.566 (10)
H23	0.216846	0.436944	0.867607	0.129*	0.566 (10)
C24	0.0619 (11)	0.4193 (3)	0.8606 (8)	0.104 (3)	0.566 (10)
H24	0.074303	0.418670	0.922528	0.125*	0.566 (10)
C25	−0.0430 (11)	0.4093 (2)	0.8014 (6)	0.090 (3)	0.566 (10)
H25	−0.100727	0.402068	0.823759	0.108*	0.566 (10)
C26	−0.0615 (11)	0.4100 (3)	0.7091 (6)	0.073 (2)	0.566 (10)
H26	−0.131292	0.403255	0.669171	0.087*	0.566 (10)
C27	−0.0007 (4)	0.48296 (11)	0.3751 (4)	0.0812 (15)	
H27A	0.013179	0.501705	0.411776	0.122*	
H27B	−0.055723	0.469828	0.390665	0.122*	
H27C	−0.030258	0.488795	0.312612	0.122*	
C28	0.1839 (4)	0.47542 (10)	0.3434 (3)	0.0586 (11)	
C29	0.2027 (6)	0.50751 (12)	0.3317 (4)	0.0901 (18)	
H29	0.162559	0.522855	0.352467	0.108*	
C30	0.2813 (7)	0.51618 (15)	0.2891 (4)	0.113 (2)	
H30	0.293604	0.537552	0.281054	0.136*	
C31	0.3408 (6)	0.49438 (18)	0.2589 (4)	0.107 (2)	
H31	0.394705	0.500724	0.231459	0.128*	
C32	0.3219 (5)	0.46320 (14)	0.2685 (4)	0.0903 (17)	
H32	0.362144	0.448145	0.246837	0.108*	
C33	0.2442 (4)	0.45371 (11)	0.3099 (3)	0.0692 (13)	
H33	0.231681	0.432208	0.315557	0.083*	
C34	0.2921 (5)	0.48750 (10)	0.5680 (4)	0.0888 (17)	
H34A	0.226485	0.492218	0.587097	0.133*	
H34B	0.293494	0.501575	0.520006	0.133*	
H34C	0.361292	0.490171	0.617880	0.133*	
C35	0.3906 (4)	0.43815 (9)	0.5492 (3)	0.0551 (10)	
C36	0.4730 (5)	0.44964 (13)	0.5137 (4)	0.0835 (15)	
H36	0.459842	0.468195	0.479894	0.100*	
C37	0.5769 (5)	0.43299 (18)	0.5291 (5)	0.112 (2)	
H37	0.632723	0.440743	0.505487	0.134*	
C38	0.5972 (5)	0.40600 (15)	0.5775 (5)	0.106 (2)	
H38	0.666582	0.395285	0.587785	0.127*	
C39	0.5164 (5)	0.39483 (12)	0.6105 (4)	0.0897 (18)	
H39	0.529842	0.375939	0.642704	0.108*	
C40	0.4136 (4)	0.41046 (10)	0.5981 (3)	0.0666 (12)	
H40	0.359587	0.402268	0.622954	0.080*	
C41	0.3684 (2)	0.29128 (6)	0.53707 (17)	0.0500 (9)	
C46	0.4646 (2)	0.29880 (7)	0.5116 (2)	0.0720 (13)	
H46	0.508373	0.316566	0.535733	0.086*	
C45	0.4954 (3)	0.27976 (10)	0.4502 (2)	0.097 (2)	
H45	0.559769	0.284794	0.433180	0.117*	
C44	0.4300 (4)	0.25321 (9)	0.4142 (2)	0.095 (2)	
H44	0.450603	0.240472	0.373076	0.114*	
C43	0.3338 (3)	0.24569 (6)	0.4396 (2)	0.0822 (16)	
H43	0.290040	0.227922	0.415524	0.099*	
C42	0.3030 (2)	0.26472 (6)	0.5011 (2)	0.0619 (11)	
H42	0.238643	0.259693	0.518078	0.074*	
C47	0.1695 (4)	0.34395 (13)	0.7563 (4)	0.0796 (15)	
H47A	0.202804	0.332059	0.810680	0.119*	
H47B	0.135822	0.363029	0.770168	0.119*	
H47C	0.228374	0.349236	0.729899	0.119*	
C48	−0.0333 (3)	0.32679 (9)	0.6974 (3)	0.0494 (9)	
C49	−0.0540 (4)	0.32713 (12)	0.7796 (3)	0.0724 (13)	
H49	0.007602	0.326656	0.832778	0.087*	
C50	−0.1649 (5)	0.32817 (13)	0.7834 (4)	0.0881 (18)	
H50	−0.177511	0.327955	0.839206	0.106*	
C51	−0.2559 (5)	0.32950 (13)	0.7070 (5)	0.0880 (17)	
H51	−0.330661	0.330433	0.710034	0.106*	
C52	−0.2367 (4)	0.32944 (12)	0.6257 (4)	0.0796 (15)	
H52	−0.298956	0.330068	0.572921	0.096*	
C53	−0.1264 (3)	0.32847 (11)	0.6202 (3)	0.0609 (11)	
H53	−0.114814	0.328955	0.564068	0.073*	
C54	0.1050 (4)	0.25407 (10)	0.7293 (3)	0.0606 (11)	
H54A	0.111391	0.270699	0.772554	0.091*	
H54B	0.179822	0.245223	0.736902	0.091*	
H54C	0.054722	0.237855	0.738422	0.091*	
C55	0.0178 (3)	0.24399 (9)	0.5675 (3)	0.0476 (9)	
C56	0.0768 (4)	0.21643 (11)	0.5693 (3)	0.0686 (12)	
H56	0.143971	0.212639	0.616564	0.082*	
C57	0.0383 (6)	0.19425 (14)	0.5025 (4)	0.102 (2)	
H57	0.079199	0.175541	0.504696	0.123*	
C58	−0.0584 (7)	0.19952 (16)	0.4338 (5)	0.106 (2)	
H58	−0.083021	0.184567	0.387985	0.127*	
C59	−0.1203 (5)	0.22615 (16)	0.4302 (4)	0.098 (2)	
H59	−0.187795	0.229374	0.382725	0.118*	
C60	−0.0821 (4)	0.24920 (12)	0.4990 (3)	0.0752 (14)	
H60	−0.124409	0.267603	0.497580	0.090*	
C61	0.0147 (3)	0.28047 (9)	0.3229 (3)	0.0513 (10)	
H61	−0.039745	0.282937	0.352804	0.062*	
C62	0.0086 (4)	0.25364 (10)	0.2700 (3)	0.0646 (12)	
H62	−0.047729	0.238366	0.265593	0.077*	
C63	0.0863 (4)	0.25026 (10)	0.2250 (3)	0.0667 (12)	
H63	0.083168	0.232540	0.188912	0.080*	
C64	0.1706 (4)	0.27301 (9)	0.2323 (3)	0.0527 (10)	
C65	0.1723 (3)	0.29913 (8)	0.2882 (2)	0.0410 (8)	
C66	0.2602 (3)	0.32280 (8)	0.3012 (2)	0.0411 (8)	
C67	0.2540 (4)	0.27103 (11)	0.1862 (3)	0.0675 (13)	
H67	0.252659	0.253858	0.148614	0.081*	
C68	0.3341 (4)	0.29336 (12)	0.1960 (3)	0.0654 (12)	
H68	0.386027	0.291779	0.163506	0.078*	
C69	0.3421 (3)	0.31963 (10)	0.2552 (3)	0.0529 (10)	
C70	0.4295 (4)	0.34229 (12)	0.2727 (3)	0.0643 (12)	
H70	0.485326	0.341081	0.243743	0.077*	
C71	0.4331 (4)	0.36611 (12)	0.3321 (3)	0.0668 (12)	
H71	0.491521	0.381151	0.344738	0.080*	
C72	0.3477 (3)	0.36758 (10)	0.3734 (3)	0.0513 (9)	
H72	0.350245	0.384125	0.413356	0.062*	
C21A	0.0465 (16)	0.4224 (4)	0.6769 (14)	0.071 (3)	0.434 (10)
C26A	−0.0189 (15)	0.4148 (4)	0.7314 (9)	0.083 (3)	0.434 (10)
H26A	−0.094691	0.408365	0.704910	0.099*	0.434 (10)
C25A	0.0245 (15)	0.4165 (3)	0.8243 (9)	0.097 (3)	0.434 (10)
H25A	−0.020609	0.411380	0.860486	0.117*	0.434 (10)
C24A	0.1374 (15)	0.4262 (4)	0.8614 (10)	0.104 (3)	0.434 (10)
H24A	0.168715	0.427479	0.923817	0.124*	0.434 (10)
C23A	0.2057 (15)	0.4341 (3)	0.8077 (8)	0.108 (3)	0.434 (10)
H23A	0.281472	0.440605	0.834370	0.130*	0.434 (10)
C22A	0.1601 (16)	0.4321 (4)	0.7136 (9)	0.090 (3)	0.434 (10)
H22A	0.204549	0.437206	0.676840	0.108*	0.434 (10)
C15A	−0.1797 (13)	0.4014 (4)	0.0492 (9)	0.071 (2)	0.445 (17)
C20A	−0.2576 (15)	0.3789 (4)	0.0009 (12)	0.082 (3)	0.445 (17)
H20A	−0.269592	0.360404	0.029188	0.098*	0.445 (17)
C19A	−0.3177 (13)	0.3839 (4)	−0.0897 (11)	0.095 (3)	0.445 (17)
H19A	−0.369885	0.368863	−0.122003	0.113*	0.445 (17)
C18A	−0.2999 (14)	0.4115 (3)	−0.1320 (8)	0.104 (3)	0.445 (17)
H18A	−0.340084	0.414929	−0.192586	0.125*	0.445 (17)
C17A	−0.2219 (17)	0.4341 (3)	−0.0837 (8)	0.110 (3)	0.445 (17)
H17A	−0.209991	0.452537	−0.111978	0.132*	0.445 (17)
C16A	−0.1618 (13)	0.4290 (4)	0.0069 (8)	0.094 (3)	0.445 (17)
H16A	−0.109697	0.444079	0.039214	0.113*	0.445 (17)
C1A	−0.291 (2)	0.3812 (4)	0.2667 (15)	0.051 (2)	0.50 (3)
C6A	−0.2696 (17)	0.4040 (4)	0.3311 (14)	0.059 (2)	0.50 (3)
H6A	−0.216818	0.400203	0.387686	0.071*	0.50 (3)
C5A	−0.3249 (16)	0.4326 (4)	0.3136 (14)	0.066 (2)	0.50 (3)
H5A	−0.309091	0.448419	0.357298	0.080*	0.50 (3)
C4A	−0.4013 (14)	0.4374 (4)	0.2331 (14)	0.068 (3)	0.50 (3)
H4A	−0.436112	0.457151	0.220362	0.082*	0.50 (3)
C3A	−0.4318 (15)	0.4140 (4)	0.1668 (13)	0.069 (3)	0.50 (3)
H3A	−0.489562	0.417491	0.112337	0.083*	0.50 (3)
C2A	−0.372 (2)	0.3851 (4)	0.1848 (14)	0.062 (2)	0.50 (3)
H2A	−0.388345	0.368995	0.141959	0.074*	0.50 (3)

### Atomic displacement parameters (Å^2^)

**Table:**

	*U*^11^	*U*^22^	*U*^33^	*U*^12^	*U*^13^	*U*^23^
La1	0.03289 (10)	0.02525 (10)	0.03624 (11)	0.00223 (8)	0.01132 (8)	−0.00094 (8)
S1	0.0369 (5)	0.0413 (5)	0.0531 (6)	−0.0020 (4)	0.0089 (4)	−0.0040 (4)
S2	0.0538 (6)	0.0428 (5)	0.0527 (6)	0.0047 (4)	0.0232 (5)	−0.0101 (4)
S3	0.0305 (4)	0.0448 (5)	0.0506 (5)	0.0031 (4)	0.0085 (4)	0.0091 (4)
P1	0.0468 (5)	0.0455 (5)	0.0374 (5)	0.0072 (4)	0.0104 (4)	−0.0001 (4)
P2	0.0597 (6)	0.0269 (5)	0.0628 (6)	0.0005 (4)	0.0270 (5)	−0.0064 (4)
P3	0.0339 (5)	0.0373 (5)	0.0417 (5)	0.0020 (4)	0.0113 (4)	0.0071 (4)
O1	0.0478 (14)	0.0413 (14)	0.0395 (13)	−0.0019 (11)	0.0110 (11)	0.0003 (11)
O2	0.0473 (14)	0.0270 (12)	0.0589 (16)	−0.0004 (10)	0.0217 (12)	−0.0065 (11)
O3	0.0356 (12)	0.0389 (13)	0.0425 (13)	−0.0003 (10)	0.0111 (11)	0.0057 (10)
O4	0.0507 (16)	0.0509 (16)	0.078 (2)	−0.0143 (13)	0.0146 (15)	−0.0070 (14)
O5	0.0393 (14)	0.0527 (15)	0.0485 (15)	0.0005 (12)	0.0110 (12)	−0.0003 (12)
O6	0.0582 (19)	0.100 (3)	0.086 (2)	0.0177 (18)	0.0180 (17)	−0.026 (2)
O7	0.0659 (17)	0.0373 (14)	0.0642 (17)	−0.0069 (12)	0.0358 (15)	−0.0131 (12)
O8	0.0359 (14)	0.0627 (17)	0.0627 (17)	−0.0036 (13)	0.0027 (12)	0.0090 (14)
O9	0.0346 (13)	0.0367 (13)	0.0556 (15)	−0.0004 (10)	0.0092 (11)	0.0087 (11)
N1	0.0459 (18)	0.0477 (18)	0.0462 (18)	0.0001 (15)	0.0069 (14)	−0.0087 (15)
N2	0.102 (3)	0.0347 (18)	0.102 (3)	−0.0026 (19)	0.069 (3)	−0.0153 (18)
N3	0.0335 (15)	0.0462 (17)	0.0520 (18)	0.0058 (13)	0.0121 (14)	0.0142 (14)
N4	0.055 (2)	0.063 (2)	0.0438 (18)	0.0096 (17)	0.0137 (16)	−0.0091 (16)
N5	0.064 (2)	0.0495 (19)	0.0467 (19)	0.0114 (16)	0.0126 (16)	0.0059 (15)
N6	0.062 (2)	0.0361 (17)	0.073 (2)	0.0105 (15)	0.0307 (19)	0.0024 (16)
N7	0.068 (2)	0.0290 (16)	0.073 (2)	−0.0048 (16)	0.0157 (19)	−0.0116 (15)
N8	0.0380 (16)	0.0492 (18)	0.0479 (18)	0.0010 (14)	0.0123 (14)	−0.0002 (14)
N9	0.0407 (16)	0.0418 (17)	0.0419 (17)	−0.0041 (13)	0.0089 (13)	0.0083 (13)
N10	0.0463 (17)	0.0323 (15)	0.0506 (18)	0.0006 (13)	0.0167 (15)	−0.0064 (13)
N11	0.0360 (16)	0.0400 (16)	0.0474 (17)	0.0050 (13)	0.0155 (13)	−0.0001 (13)
C1	0.039 (4)	0.051 (4)	0.065 (5)	0.001 (3)	0.013 (4)	−0.001 (4)
C2	0.052 (4)	0.053 (5)	0.071 (5)	0.007 (4)	0.008 (4)	−0.004 (4)
C3	0.058 (4)	0.058 (5)	0.075 (6)	0.010 (4)	−0.001 (4)	−0.008 (4)
C4	0.061 (5)	0.055 (4)	0.078 (6)	0.016 (4)	0.003 (5)	−0.017 (4)
C5	0.054 (5)	0.061 (4)	0.072 (6)	0.013 (4)	−0.002 (4)	−0.008 (4)
C6	0.047 (5)	0.052 (4)	0.067 (5)	0.015 (3)	0.004 (4)	−0.008 (4)
C7	0.086 (4)	0.072 (3)	0.063 (3)	0.011 (3)	0.017 (3)	−0.025 (2)
C8	0.058 (3)	0.089 (3)	0.037 (2)	0.019 (2)	0.0115 (19)	−0.001 (2)
C9	0.075 (3)	0.099 (4)	0.071 (3)	−0.001 (3)	0.039 (3)	−0.003 (3)
C10	0.085 (4)	0.151 (6)	0.086 (4)	−0.012 (4)	0.043 (3)	0.004 (4)
C11	0.084 (5)	0.201 (9)	0.077 (4)	0.008 (5)	0.043 (4)	0.006 (5)
C12	0.096 (5)	0.196 (8)	0.066 (4)	0.035 (5)	0.039 (4)	−0.007 (5)
C13	0.085 (4)	0.124 (5)	0.053 (3)	0.028 (3)	0.023 (3)	−0.008 (3)
C14	0.097 (4)	0.047 (3)	0.078 (3)	0.011 (3)	0.022 (3)	0.004 (2)
C15	0.074 (4)	0.083 (4)	0.047 (3)	0.020 (4)	0.021 (3)	0.020 (3)
C20	0.081 (4)	0.103 (5)	0.055 (4)	0.027 (4)	0.007 (3)	0.012 (4)
C19	0.097 (4)	0.118 (5)	0.059 (4)	0.020 (4)	0.005 (4)	0.016 (4)
C18	0.105 (5)	0.132 (5)	0.066 (4)	0.006 (5)	0.002 (4)	0.032 (4)
C17	0.109 (6)	0.123 (5)	0.075 (4)	0.010 (5)	−0.001 (5)	0.044 (4)
C16	0.097 (5)	0.104 (4)	0.068 (4)	0.012 (5)	0.001 (4)	0.036 (4)
C21	0.096 (5)	0.059 (4)	0.050 (3)	0.010 (4)	0.025 (4)	−0.010 (3)
C22	0.117 (6)	0.096 (4)	0.056 (4)	−0.001 (4)	0.024 (4)	−0.013 (4)
C23	0.135 (6)	0.119 (4)	0.064 (5)	−0.006 (5)	0.026 (4)	−0.011 (4)
C24	0.131 (6)	0.111 (5)	0.066 (4)	0.001 (5)	0.024 (4)	−0.012 (4)
C25	0.120 (6)	0.092 (5)	0.066 (5)	0.007 (5)	0.041 (4)	−0.005 (4)
C26	0.105 (6)	0.069 (4)	0.053 (4)	0.009 (4)	0.038 (4)	−0.010 (3)
C27	0.081 (3)	0.057 (3)	0.109 (4)	0.028 (3)	0.033 (3)	0.004 (3)
C28	0.073 (3)	0.044 (2)	0.061 (3)	0.009 (2)	0.024 (2)	0.0064 (19)
C29	0.143 (5)	0.052 (3)	0.093 (4)	0.007 (3)	0.062 (4)	0.013 (3)
C30	0.188 (7)	0.068 (4)	0.108 (5)	−0.018 (4)	0.081 (5)	0.017 (3)
C31	0.131 (6)	0.113 (5)	0.100 (5)	−0.008 (5)	0.069 (4)	0.013 (4)
C32	0.107 (4)	0.084 (4)	0.097 (4)	0.012 (3)	0.058 (4)	0.014 (3)
C33	0.082 (3)	0.050 (3)	0.086 (3)	0.011 (2)	0.041 (3)	0.010 (2)
C34	0.109 (4)	0.039 (2)	0.111 (4)	−0.013 (3)	0.025 (4)	−0.032 (3)
C35	0.059 (3)	0.041 (2)	0.061 (3)	−0.0118 (19)	0.012 (2)	−0.0073 (18)
C36	0.079 (4)	0.073 (3)	0.098 (4)	−0.018 (3)	0.028 (3)	0.012 (3)
C37	0.075 (4)	0.123 (6)	0.147 (6)	−0.034 (4)	0.050 (4)	−0.022 (5)
C38	0.060 (3)	0.073 (4)	0.167 (7)	−0.006 (3)	0.010 (4)	−0.018 (4)
C39	0.067 (3)	0.058 (3)	0.118 (5)	−0.005 (3)	−0.008 (3)	0.004 (3)
C40	0.064 (3)	0.049 (3)	0.078 (3)	−0.013 (2)	0.009 (2)	−0.003 (2)
C41	0.045 (2)	0.053 (2)	0.053 (2)	0.0169 (18)	0.0167 (18)	0.0167 (19)
C46	0.053 (3)	0.094 (4)	0.074 (3)	0.016 (3)	0.027 (2)	0.014 (3)
C45	0.083 (4)	0.134 (6)	0.089 (4)	0.051 (4)	0.049 (3)	0.029 (4)
C44	0.126 (5)	0.088 (4)	0.079 (4)	0.061 (4)	0.043 (4)	0.017 (3)
C43	0.111 (4)	0.058 (3)	0.074 (3)	0.032 (3)	0.023 (3)	0.008 (3)
C42	0.072 (3)	0.046 (2)	0.069 (3)	0.015 (2)	0.024 (2)	0.009 (2)
C47	0.067 (3)	0.085 (3)	0.080 (3)	−0.004 (3)	0.014 (3)	−0.036 (3)
C48	0.050 (2)	0.045 (2)	0.057 (2)	0.0090 (18)	0.0228 (19)	0.0061 (18)
C49	0.075 (3)	0.085 (3)	0.066 (3)	0.023 (3)	0.035 (3)	0.016 (3)
C50	0.099 (4)	0.097 (4)	0.094 (4)	0.035 (3)	0.068 (4)	0.031 (3)
C51	0.076 (4)	0.086 (4)	0.124 (5)	0.016 (3)	0.063 (4)	0.021 (4)
C52	0.046 (3)	0.089 (4)	0.106 (4)	0.011 (3)	0.027 (3)	0.004 (3)
C53	0.045 (2)	0.074 (3)	0.066 (3)	0.008 (2)	0.020 (2)	0.005 (2)
C54	0.074 (3)	0.055 (2)	0.045 (2)	−0.013 (2)	0.007 (2)	0.0161 (19)
C55	0.045 (2)	0.048 (2)	0.048 (2)	−0.0138 (18)	0.0132 (18)	0.0088 (17)
C56	0.082 (3)	0.052 (3)	0.066 (3)	−0.007 (2)	0.015 (3)	−0.006 (2)
C57	0.139 (6)	0.065 (4)	0.094 (5)	−0.010 (4)	0.024 (4)	−0.017 (3)
C58	0.136 (6)	0.078 (4)	0.088 (5)	−0.045 (4)	0.013 (4)	−0.008 (3)
C59	0.087 (4)	0.109 (5)	0.069 (3)	−0.043 (4)	−0.019 (3)	0.020 (3)
C60	0.062 (3)	0.077 (3)	0.071 (3)	−0.024 (3)	−0.001 (2)	0.018 (3)
C61	0.057 (2)	0.038 (2)	0.060 (2)	−0.0045 (18)	0.020 (2)	−0.0070 (18)
C62	0.072 (3)	0.045 (2)	0.073 (3)	−0.015 (2)	0.018 (2)	−0.019 (2)
C63	0.081 (3)	0.047 (2)	0.068 (3)	0.003 (2)	0.016 (3)	−0.021 (2)
C64	0.059 (2)	0.046 (2)	0.052 (2)	0.0152 (19)	0.014 (2)	−0.0069 (18)
C65	0.044 (2)	0.0394 (19)	0.0393 (19)	0.0107 (16)	0.0125 (16)	−0.0010 (15)
C66	0.0388 (19)	0.045 (2)	0.0416 (19)	0.0129 (16)	0.0157 (16)	0.0020 (16)
C67	0.077 (3)	0.064 (3)	0.063 (3)	0.017 (3)	0.024 (2)	−0.019 (2)
C68	0.063 (3)	0.084 (3)	0.056 (3)	0.025 (3)	0.029 (2)	−0.007 (2)
C69	0.047 (2)	0.063 (3)	0.051 (2)	0.016 (2)	0.0197 (19)	0.004 (2)
C70	0.048 (2)	0.080 (3)	0.073 (3)	0.010 (2)	0.031 (2)	0.004 (3)
C71	0.045 (2)	0.074 (3)	0.086 (3)	−0.006 (2)	0.026 (2)	0.002 (3)
C72	0.042 (2)	0.049 (2)	0.065 (3)	−0.0010 (18)	0.0197 (19)	−0.0051 (19)
C21A	0.102 (6)	0.063 (4)	0.051 (4)	0.012 (4)	0.027 (4)	−0.012 (4)
C26A	0.114 (6)	0.082 (4)	0.056 (4)	0.011 (4)	0.031 (4)	−0.010 (4)
C25A	0.128 (6)	0.101 (5)	0.061 (5)	0.003 (5)	0.028 (5)	−0.013 (4)
C24A	0.134 (6)	0.116 (5)	0.061 (5)	−0.006 (5)	0.031 (5)	−0.006 (4)
C23A	0.132 (7)	0.119 (5)	0.067 (5)	−0.009 (6)	0.024 (5)	−0.013 (5)
C22A	0.119 (6)	0.095 (5)	0.051 (5)	−0.002 (5)	0.021 (4)	−0.013 (4)
C15A	0.076 (4)	0.086 (4)	0.051 (4)	0.020 (4)	0.017 (4)	0.019 (4)
C20A	0.083 (4)	0.098 (5)	0.054 (4)	0.024 (4)	0.007 (4)	0.018 (4)
C19A	0.096 (4)	0.113 (5)	0.059 (4)	0.019 (5)	0.002 (4)	0.020 (4)
C18A	0.108 (5)	0.128 (5)	0.062 (4)	0.006 (5)	0.006 (5)	0.032 (4)
C17A	0.108 (6)	0.127 (5)	0.077 (4)	0.006 (5)	0.003 (5)	0.038 (4)
C16A	0.093 (5)	0.109 (5)	0.067 (4)	0.010 (5)	0.006 (5)	0.033 (4)
C1A	0.039 (4)	0.050 (4)	0.063 (4)	0.002 (3)	0.015 (4)	−0.003 (3)
C6A	0.049 (5)	0.057 (4)	0.065 (5)	0.009 (4)	0.009 (4)	−0.005 (4)
C5A	0.056 (5)	0.061 (4)	0.069 (6)	0.015 (4)	0.000 (4)	−0.012 (4)
C4A	0.061 (5)	0.058 (4)	0.076 (6)	0.015 (4)	0.008 (5)	−0.012 (4)
C3A	0.060 (4)	0.056 (5)	0.079 (6)	0.013 (4)	0.003 (4)	−0.005 (4)
C2A	0.052 (4)	0.054 (4)	0.071 (5)	0.009 (4)	0.008 (4)	−0.002 (4)

### Geometric parameters (Å, º)

**Table:**

La1—O1	2.430 (2)	C31—C32	1.354 (8)
La1—O2	2.424 (2)	C32—H32	0.9300
La1—O3	2.463 (2)	C32—C33	1.364 (7)
La1—O5	2.541 (2)	C33—H33	0.9300
La1—O7	2.535 (2)	C34—H34A	0.9600
La1—O9	2.516 (2)	C34—H34B	0.9600
La1—N10	2.699 (3)	C34—H34C	0.9600
La1—N11	2.695 (3)	C35—C36	1.380 (6)
S1—O4	1.436 (3)	C35—C40	1.377 (6)
S1—O5	1.462 (3)	C36—H36	0.9300
S1—N1	1.553 (3)	C36—C37	1.406 (8)
S1—C1	1.756 (10)	C37—H37	0.9300
S1—C1A	1.778 (13)	C37—C38	1.347 (9)
S2—O6	1.432 (3)	C38—H38	0.9300
S2—O7	1.468 (2)	C38—C39	1.334 (8)
S2—N2	1.514 (4)	C39—H39	0.9300
S2—C21	1.751 (16)	C39—C40	1.379 (7)
S2—C21A	1.74 (2)	C40—H40	0.9300
S3—O8	1.436 (3)	C41—C46	1.3900
S3—O9	1.474 (2)	C41—C42	1.3900
S3—N3	1.538 (3)	C46—H46	0.9300
S3—C41	1.756 (2)	C46—C45	1.3900
P1—O1	1.494 (2)	C45—H45	0.9300
P1—N1	1.597 (3)	C45—C44	1.3900
P1—N4	1.661 (3)	C44—H44	0.9300
P1—N5	1.664 (3)	C44—C43	1.3900
P2—O2	1.493 (2)	C43—H43	0.9300
P2—N2	1.590 (4)	C43—C42	1.3900
P2—N6	1.656 (3)	C42—H42	0.9300
P2—N7	1.642 (4)	C47—H47A	0.9600
P3—O3	1.491 (2)	C47—H47B	0.9600
P3—N3	1.610 (3)	C47—H47C	0.9600
P3—N8	1.651 (3)	C48—C49	1.382 (6)
P3—N9	1.655 (3)	C48—C53	1.375 (5)
N4—C7	1.467 (6)	C49—H49	0.9300
N4—C8	1.414 (6)	C49—C50	1.376 (7)
N5—C14	1.475 (5)	C50—H50	0.9300
N5—C15	1.437 (9)	C50—C51	1.353 (8)
N5—C15A	1.372 (11)	C51—H51	0.9300
N6—C27	1.474 (5)	C51—C52	1.360 (7)
N6—C28	1.421 (5)	C52—H52	0.9300
N7—C34	1.484 (5)	C52—C53	1.378 (6)
N7—C35	1.421 (5)	C53—H53	0.9300
N8—C47	1.451 (5)	C54—H54A	0.9600
N8—C48	1.424 (5)	C54—H54B	0.9600
N9—C54	1.466 (4)	C54—H54C	0.9600
N9—C55	1.430 (5)	C55—C56	1.365 (6)
N10—C61	1.320 (4)	C55—C60	1.365 (5)
N10—C65	1.358 (4)	C56—H56	0.9300
N11—C66	1.355 (4)	C56—C57	1.371 (7)
N11—C72	1.319 (5)	C57—H57	0.9300
C1—C2	1.3900	C57—C58	1.342 (8)
C1—C6	1.3900	C58—H58	0.9300
C2—H2	0.9300	C58—C59	1.347 (9)
C2—C3	1.3900	C59—H59	0.9300
C3—H3	0.9300	C59—C60	1.416 (7)
C3—C4	1.3900	C60—H60	0.9300
C4—H4	0.9300	C61—H61	0.9300
C4—C5	1.3900	C61—C62	1.390 (5)
C5—H5	0.9300	C62—H62	0.9300
C5—C6	1.3900	C62—C63	1.354 (6)
C6—H6	0.9300	C63—H63	0.9300
C7—H7A	0.9600	C63—C64	1.387 (6)
C7—H7B	0.9600	C64—C65	1.402 (5)
C7—H7C	0.9600	C64—C67	1.421 (6)
C8—C9	1.360 (7)	C65—C66	1.435 (5)
C8—C13	1.398 (6)	C66—C69	1.408 (5)
C9—H9	0.9300	C67—H67	0.9300
C9—C10	1.385 (7)	C67—C68	1.334 (6)
C10—H10	0.9300	C68—H68	0.9300
C10—C11	1.360 (9)	C68—C69	1.427 (6)
C11—H11	0.9300	C69—C70	1.396 (6)
C11—C12	1.330 (10)	C70—H70	0.9300
C12—H12	0.9300	C70—C71	1.359 (6)
C12—C13	1.380 (9)	C71—H71	0.9300
C13—H13	0.9300	C71—C72	1.389 (6)
C14—H14A	0.9600	C72—H72	0.9300
C14—H14B	0.9600	C21A—C26A	1.373 (9)
C14—H14C	0.9600	C21A—C22A	1.389 (9)
C15—C20	1.3900	C26A—H26A	0.9300
C15—C16	1.3900	C26A—C25A	1.379 (9)
C20—H20	0.9300	C25A—H25A	0.9300
C20—C19	1.3900	C25A—C24A	1.380 (9)
C19—H19	0.9300	C24A—H24A	0.9300
C19—C18	1.3900	C24A—C23A	1.396 (9)
C18—H18	0.9300	C23A—H23A	0.9300
C18—C17	1.3900	C23A—C22A	1.399 (9)
C17—H17	0.9300	C22A—H22A	0.9300
C17—C16	1.3900	C15A—C20A	1.3900
C16—H16	0.9300	C15A—C16A	1.3900
C21—C22	1.361 (9)	C20A—H20A	0.9300
C21—C26	1.379 (8)	C20A—C19A	1.3900
C22—H22	0.9300	C19A—H19A	0.9300
C22—C23	1.387 (8)	C19A—C18A	1.3900
C23—H23	0.9300	C18A—H18A	0.9300
C23—C24	1.367 (9)	C18A—C17A	1.3900
C24—H24	0.9300	C17A—H17A	0.9300
C24—C25	1.391 (9)	C17A—C16A	1.3900
C25—H25	0.9300	C16A—H16A	0.9300
C25—C26	1.386 (8)	C1A—C6A	1.358 (12)
C26—H26	0.9300	C1A—C2A	1.360 (12)
C27—H27A	0.9600	C6A—H6A	0.9300
C27—H27B	0.9600	C6A—C5A	1.370 (14)
C27—H27C	0.9600	C5A—H5A	0.9300
C28—C29	1.396 (6)	C5A—C4A	1.324 (14)
C28—C33	1.377 (6)	C4A—H4A	0.9300
C29—H29	0.9300	C4A—C3A	1.394 (14)
C29—C30	1.378 (8)	C3A—H3A	0.9300
C30—H30	0.9300	C3A—C2A	1.405 (14)
C30—C31	1.346 (8)	C2A—H2A	0.9300
C31—H31	0.9300		
			
O1—La1—O3	138.81 (8)	C33—C28—C29	117.9 (5)
O1—La1—O5	70.70 (8)	C28—C29—H29	120.4
O1—La1—O7	112.43 (9)	C30—C29—C28	119.3 (5)
O1—La1—O9	141.64 (8)	C30—C29—H29	120.4
O1—La1—N10	75.35 (8)	C29—C30—H30	119.3
O1—La1—N11	71.91 (8)	C31—C30—C29	121.4 (6)
O2—La1—O1	79.32 (8)	C31—C30—H30	119.3
O2—La1—O3	140.85 (8)	C30—C31—H31	120.1
O2—La1—O5	122.18 (8)	C30—C31—C32	119.8 (6)
O2—La1—O7	70.52 (8)	C32—C31—H31	120.1
O2—La1—O9	81.65 (8)	C31—C32—H32	119.8
O2—La1—N10	136.74 (8)	C31—C32—C33	120.4 (5)
O2—La1—N11	78.48 (8)	C33—C32—H32	119.8
O3—La1—O5	76.66 (8)	C28—C33—H33	119.4
O3—La1—O7	83.00 (8)	C32—C33—C28	121.2 (5)
O3—La1—O9	70.60 (7)	C32—C33—H33	119.4
O3—La1—N10	75.41 (9)	N7—C34—H34A	109.5
O3—La1—N11	116.22 (8)	N7—C34—H34B	109.5
O5—La1—N10	81.14 (9)	N7—C34—H34C	109.5
O5—La1—N11	131.75 (8)	H34A—C34—H34B	109.5
O7—La1—O5	76.97 (8)	H34A—C34—H34C	109.5
O7—La1—N10	152.22 (9)	H34B—C34—H34C	109.5
O7—La1—N11	146.79 (9)	C36—C35—N7	121.1 (4)
O9—La1—O5	146.45 (8)	C40—C35—N7	120.7 (4)
O9—La1—O7	91.76 (9)	C40—C35—C36	118.3 (5)
O9—La1—N10	97.23 (9)	C35—C36—H36	120.4
O9—La1—N11	71.83 (8)	C35—C36—C37	119.3 (5)
N11—La1—N10	60.60 (9)	C37—C36—H36	120.4
O4—S1—O5	113.73 (17)	C36—C37—H37	119.4
O4—S1—N1	110.09 (18)	C38—C37—C36	121.2 (6)
O4—S1—C1	108.5 (9)	C38—C37—H37	119.4
O4—S1—C1A	107.5 (9)	C37—C38—H38	120.4
O5—S1—N1	112.78 (16)	C39—C38—C37	119.2 (6)
O5—S1—C1	104.2 (6)	C39—C38—H38	120.4
O5—S1—C1A	107.5 (7)	C38—C39—H39	119.0
N1—S1—C1	107.1 (9)	C38—C39—C40	121.9 (5)
N1—S1—C1A	104.7 (9)	C40—C39—H39	119.0
O6—S2—O7	112.93 (19)	C35—C40—C39	120.2 (5)
O6—S2—N2	112.7 (2)	C35—C40—H40	119.9
O6—S2—C21	100.7 (5)	C39—C40—H40	119.9
O6—S2—C21A	108.4 (6)	C46—C41—S3	118.33 (18)
O7—S2—N2	113.08 (17)	C46—C41—C42	120.0
O7—S2—C21	107.1 (5)	C42—C41—S3	121.66 (18)
O7—S2—C21A	107.2 (6)	C41—C46—H46	120.0
N2—S2—C21	109.4 (5)	C45—C46—C41	120.0
N2—S2—C21A	101.6 (6)	C45—C46—H46	120.0
O8—S3—O9	113.33 (16)	C46—C45—H45	120.0
O8—S3—N3	111.49 (17)	C46—C45—C44	120.0
O8—S3—C41	106.54 (16)	C44—C45—H45	120.0
O9—S3—N3	112.43 (15)	C45—C44—H44	120.0
O9—S3—C41	105.18 (14)	C43—C44—C45	120.0
N3—S3—C41	107.32 (16)	C43—C44—H44	120.0
O1—P1—N1	115.44 (15)	C44—C43—H43	120.0
O1—P1—N4	113.08 (16)	C44—C43—C42	120.0
O1—P1—N5	106.05 (16)	C42—C43—H43	120.0
N1—P1—N4	103.39 (18)	C41—C42—H42	120.0
N1—P1—N5	112.44 (18)	C43—C42—C41	120.0
N4—P1—N5	106.21 (17)	C43—C42—H42	120.0
O2—P2—N2	116.54 (16)	N8—C47—H47A	109.5
O2—P2—N6	113.21 (16)	N8—C47—H47B	109.5
O2—P2—N7	108.21 (16)	N8—C47—H47C	109.5
N2—P2—N6	104.24 (19)	H47A—C47—H47B	109.5
N2—P2—N7	108.4 (2)	H47A—C47—H47C	109.5
N7—P2—N6	105.63 (18)	H47B—C47—H47C	109.5
O3—P3—N3	115.74 (15)	C49—C48—N8	120.8 (4)
O3—P3—N8	113.77 (15)	C53—C48—N8	121.2 (4)
O3—P3—N9	109.94 (14)	C53—C48—C49	118.0 (4)
N3—P3—N8	105.68 (16)	C48—C49—H49	119.7
N3—P3—N9	107.60 (15)	C50—C49—C48	120.6 (5)
N8—P3—N9	103.20 (16)	C50—C49—H49	119.7
P1—O1—La1	138.87 (14)	C49—C50—H50	119.6
P2—O2—La1	141.94 (14)	C51—C50—C49	120.8 (5)
P3—O3—La1	133.87 (13)	C51—C50—H50	119.6
S1—O5—La1	140.15 (15)	C50—C51—H51	120.4
S2—O7—La1	143.59 (15)	C50—C51—C52	119.1 (5)
S3—O9—La1	138.28 (14)	C52—C51—H51	120.4
S1—N1—P1	123.3 (2)	C51—C52—H52	119.5
S2—N2—P2	131.5 (2)	C51—C52—C53	121.0 (5)
S3—N3—P3	124.93 (19)	C53—C52—H52	119.5
C7—N4—P1	120.9 (3)	C48—C53—C52	120.4 (4)
C8—N4—P1	121.6 (3)	C48—C53—H53	119.8
C8—N4—C7	117.5 (4)	C52—C53—H53	119.8
C14—N5—P1	119.2 (3)	N9—C54—H54A	109.5
C15—N5—P1	123.8 (6)	N9—C54—H54B	109.5
C15—N5—C14	116.8 (6)	N9—C54—H54C	109.5
C15A—N5—P1	123.7 (8)	H54A—C54—H54B	109.5
C15A—N5—C14	117.1 (8)	H54A—C54—H54C	109.5
C27—N6—P2	122.8 (3)	H54B—C54—H54C	109.5
C28—N6—P2	118.2 (3)	C56—C55—N9	120.7 (4)
C28—N6—C27	117.6 (4)	C56—C55—C60	119.4 (4)
C34—N7—P2	118.5 (3)	C60—C55—N9	119.9 (4)
C35—N7—P2	124.6 (3)	C55—C56—H56	119.5
C35—N7—C34	116.5 (4)	C55—C56—C57	120.9 (5)
C47—N8—P3	121.7 (3)	C57—C56—H56	119.5
C48—N8—P3	120.0 (3)	C56—C57—H57	120.0
C48—N8—C47	118.3 (3)	C58—C57—C56	120.0 (6)
C54—N9—P3	115.9 (2)	C58—C57—H57	120.0
C55—N9—P3	121.4 (2)	C57—C58—H58	119.5
C55—N9—C54	115.6 (3)	C57—C58—C59	121.0 (6)
C61—N10—La1	120.6 (2)	C59—C58—H58	119.5
C61—N10—C65	118.2 (3)	C58—C59—H59	120.2
C65—N10—La1	121.1 (2)	C58—C59—C60	119.7 (5)
C66—N11—La1	120.9 (2)	C60—C59—H59	120.2
C72—N11—La1	121.2 (2)	C55—C60—C59	119.0 (5)
C72—N11—C66	117.7 (3)	C55—C60—H60	120.5
C2—C1—S1	121.9 (10)	C59—C60—H60	120.5
C2—C1—C6	120.0	N10—C61—H61	118.3
C6—C1—S1	117.9 (10)	N10—C61—C62	123.3 (4)
C1—C2—H2	120.0	C62—C61—H61	118.3
C3—C2—C1	120.0	C61—C62—H62	120.7
C3—C2—H2	120.0	C63—C62—C61	118.5 (4)
C2—C3—H3	120.0	C63—C62—H62	120.7
C4—C3—C2	120.0	C62—C63—H63	119.7
C4—C3—H3	120.0	C62—C63—C64	120.5 (4)
C3—C4—H4	120.0	C64—C63—H63	119.7
C3—C4—C5	120.0	C63—C64—C65	117.6 (4)
C5—C4—H4	120.0	C63—C64—C67	123.3 (4)
C4—C5—H5	120.0	C65—C64—C67	119.1 (4)
C4—C5—C6	120.0	N10—C65—C64	121.9 (3)
C6—C5—H5	120.0	N10—C65—C66	118.0 (3)
C1—C6—H6	120.0	C64—C65—C66	120.1 (3)
C5—C6—C1	120.0	N11—C66—C65	118.6 (3)
C5—C6—H6	120.0	N11—C66—C69	122.5 (3)
N4—C7—H7A	109.5	C69—C66—C65	118.8 (3)
N4—C7—H7B	109.5	C64—C67—H67	119.4
N4—C7—H7C	109.5	C68—C67—C64	121.2 (4)
H7A—C7—H7B	109.5	C68—C67—H67	119.4
H7A—C7—H7C	109.5	C67—C68—H68	119.2
H7B—C7—H7C	109.5	C67—C68—C69	121.5 (4)
C9—C8—N4	123.2 (4)	C69—C68—H68	119.2
C9—C8—C13	116.7 (5)	C66—C69—C68	119.2 (4)
C13—C8—N4	120.1 (5)	C70—C69—C66	117.2 (4)
C8—C9—H9	119.5	C70—C69—C68	123.6 (4)
C8—C9—C10	121.0 (5)	C69—C70—H70	120.0
C10—C9—H9	119.5	C71—C70—C69	120.0 (4)
C9—C10—H10	119.3	C71—C70—H70	120.0
C11—C10—C9	121.3 (7)	C70—C71—H71	120.6
C11—C10—H10	119.3	C70—C71—C72	118.7 (4)
C10—C11—H11	120.6	C72—C71—H71	120.6
C12—C11—C10	118.7 (7)	N11—C72—C71	123.8 (4)
C12—C11—H11	120.6	N11—C72—H72	118.1
C11—C12—H12	119.3	C71—C72—H72	118.1
C11—C12—C13	121.3 (7)	C26A—C21A—S2	123.4 (13)
C13—C12—H12	119.3	C26A—C21A—C22A	120.9 (17)
C8—C13—H13	119.5	C22A—C21A—S2	115.7 (12)
C12—C13—C8	121.0 (6)	C21A—C26A—H26A	119.0
C12—C13—H13	119.5	C21A—C26A—C25A	122.1 (16)
N5—C14—H14A	109.5	C25A—C26A—H26A	119.0
N5—C14—H14B	109.5	C26A—C25A—H25A	121.3
N5—C14—H14C	109.5	C26A—C25A—C24A	117.4 (15)
H14A—C14—H14B	109.5	C24A—C25A—H25A	121.3
H14A—C14—H14C	109.5	C25A—C24A—H24A	119.1
H14B—C14—H14C	109.5	C25A—C24A—C23A	121.8 (15)
C20—C15—N5	118.8 (8)	C23A—C24A—H24A	119.1
C20—C15—C16	120.0	C24A—C23A—H23A	120.1
C16—C15—N5	121.2 (8)	C24A—C23A—C22A	119.9 (15)
C15—C20—H20	120.0	C22A—C23A—H23A	120.1
C15—C20—C19	120.0	C21A—C22A—C23A	118.0 (16)
C19—C20—H20	120.0	C21A—C22A—H22A	121.0
C20—C19—H19	120.0	C23A—C22A—H22A	121.0
C18—C19—C20	120.0	N5—C15A—C20A	125.0 (11)
C18—C19—H19	120.0	N5—C15A—C16A	115.0 (11)
C19—C18—H18	120.0	C20A—C15A—C16A	120.0
C17—C18—C19	120.0	C15A—C20A—H20A	120.0
C17—C18—H18	120.0	C19A—C20A—C15A	120.0
C18—C17—H17	120.0	C19A—C20A—H20A	120.0
C18—C17—C16	120.0	C20A—C19A—H19A	120.0
C16—C17—H17	120.0	C20A—C19A—C18A	120.0
C15—C16—H16	120.0	C18A—C19A—H19A	120.0
C17—C16—C15	120.0	C19A—C18A—H18A	120.0
C17—C16—H16	120.0	C17A—C18A—C19A	120.0
C22—C21—S2	123.4 (10)	C17A—C18A—H18A	120.0
C22—C21—C26	120.3 (13)	C18A—C17A—H17A	120.0
C26—C21—S2	116.2 (9)	C18A—C17A—C16A	120.0
C21—C22—H22	119.5	C16A—C17A—H17A	120.0
C21—C22—C23	121.1 (12)	C15A—C16A—H16A	120.0
C23—C22—H22	119.5	C17A—C16A—C15A	120.0
C22—C23—H23	120.5	C17A—C16A—H16A	120.0
C24—C23—C22	119.0 (12)	C6A—C1A—S1	123.7 (13)
C24—C23—H23	120.5	C6A—C1A—C2A	121.6 (9)
C23—C24—H24	119.8	C2A—C1A—S1	114.7 (12)
C23—C24—C25	120.3 (12)	C1A—C6A—H6A	119.7
C25—C24—H24	119.8	C1A—C6A—C5A	120.6 (10)
C24—C25—H25	120.0	C5A—C6A—H6A	119.7
C26—C25—C24	119.9 (11)	C6A—C5A—H5A	120.6
C26—C25—H25	120.0	C4A—C5A—C6A	118.7 (10)
C21—C26—C25	119.3 (11)	C4A—C5A—H5A	120.6
C21—C26—H26	120.4	C5A—C4A—H4A	118.6
C25—C26—H26	120.4	C5A—C4A—C3A	122.8 (11)
N6—C27—H27A	109.5	C3A—C4A—H4A	118.6
N6—C27—H27B	109.5	C4A—C3A—H3A	121.1
N6—C27—H27C	109.5	C4A—C3A—C2A	117.8 (10)
H27A—C27—H27B	109.5	C2A—C3A—H3A	121.1
H27A—C27—H27C	109.5	C1A—C2A—C3A	118.2 (10)
H27B—C27—H27C	109.5	C1A—C2A—H2A	120.9
C29—C28—N6	121.2 (4)	C3A—C2A—H2A	120.9
C33—C28—N6	120.9 (4)		
			
La1—N10—C61—C62	177.1 (3)	N8—C48—C49—C50	−178.8 (4)
La1—N10—C65—C64	−175.9 (3)	N8—C48—C53—C52	178.7 (4)
La1—N10—C65—C66	5.5 (4)	N9—P3—O3—La1	−174.03 (17)
La1—N11—C66—C65	−8.6 (4)	N9—P3—N3—S3	150.9 (2)
La1—N11—C66—C69	173.4 (3)	N9—P3—N8—C47	126.7 (4)
La1—N11—C72—C71	−174.2 (3)	N9—P3—N8—C48	−51.4 (3)
S1—C1—C2—C3	−175.0 (18)	N9—C55—C56—C57	179.5 (4)
S1—C1—C6—C5	175.2 (17)	N9—C55—C60—C59	−179.9 (4)
S1—C1A—C6A—C5A	175.3 (19)	N10—C61—C62—C63	−1.3 (7)
S1—C1A—C2A—C3A	−177.0 (18)	N10—C65—C66—N11	2.1 (5)
S2—C21—C22—C23	−179.3 (10)	N10—C65—C66—C69	−179.9 (3)
S2—C21—C26—C25	179.4 (9)	N11—C66—C69—C68	179.1 (4)
S2—C21A—C26A—C25A	−179.8 (14)	N11—C66—C69—C70	0.9 (6)
S2—C21A—C22A—C23A	179.7 (13)	C1—S1—O5—La1	−101.7 (10)
S3—C41—C46—C45	−178.4 (2)	C1—S1—N1—P1	68.0 (8)
S3—C41—C42—C43	178.3 (2)	C1—C2—C3—C4	0.0
P1—N4—C8—C9	−0.3 (6)	C2—C1—C6—C5	0.0
P1—N4—C8—C13	177.4 (3)	C2—C3—C4—C5	0.0
P1—N5—C15—C20	13.4 (9)	C3—C4—C5—C6	0.0
P1—N5—C15—C16	−168.9 (6)	C4—C5—C6—C1	0.0
P1—N5—C15A—C20A	32.4 (12)	C6—C1—C2—C3	0.0
P1—N5—C15A—C16A	−147.3 (8)	C7—N4—C8—C9	179.4 (4)
P2—N6—C28—C29	−126.1 (4)	C7—N4—C8—C13	−2.9 (6)
P2—N6—C28—C33	51.6 (6)	C8—C9—C10—C11	−0.7 (9)
P2—N7—C35—C36	113.6 (4)	C9—C8—C13—C12	0.4 (8)
P2—N7—C35—C40	−66.0 (5)	C9—C10—C11—C12	−0.3 (11)
P3—N8—C48—C49	136.6 (4)	C10—C11—C12—C13	1.4 (12)
P3—N8—C48—C53	−44.2 (5)	C11—C12—C13—C8	−1.5 (10)
P3—N9—C55—C56	110.1 (4)	C13—C8—C9—C10	0.6 (8)
P3—N9—C55—C60	−71.8 (4)	C14—N5—C15—C20	−162.4 (6)
O1—P1—N1—S1	34.3 (3)	C14—N5—C15—C16	15.2 (11)
O1—P1—N4—C7	128.0 (3)	C14—N5—C15A—C20A	−149.0 (7)
O1—P1—N4—C8	−52.3 (4)	C14—N5—C15A—C16A	31.3 (13)
O1—P1—N5—C14	−14.2 (4)	C15—C20—C19—C18	0.0
O1—P1—N5—C15	170.0 (7)	C20—C15—C16—C17	0.0
O1—P1—N5—C15A	164.4 (9)	C20—C19—C18—C17	0.0
O2—P2—N2—S2	−5.2 (5)	C19—C18—C17—C16	0.0
O2—P2—N6—C27	121.4 (3)	C18—C17—C16—C15	0.0
O2—P2—N6—C28	−72.4 (3)	C16—C15—C20—C19	0.0
O2—P2—N7—C34	176.2 (3)	C21—S2—O7—La1	−139.0 (5)
O2—P2—N7—C35	4.2 (4)	C21—S2—N2—P2	125.8 (6)
O3—P3—N3—S3	27.6 (3)	C21—C22—C23—C24	0.1 (3)
O3—P3—N8—C47	−114.3 (4)	C22—C21—C26—C25	0.0 (6)
O3—P3—N8—C48	67.7 (3)	C22—C23—C24—C25	−0.4 (6)
O3—P3—N9—C54	178.8 (3)	C23—C24—C25—C26	0.4 (8)
O3—P3—N9—C55	29.6 (3)	C24—C25—C26—C21	−0.2 (8)
O4—S1—O5—La1	140.4 (2)	C26—C21—C22—C23	0.0 (3)
O4—S1—N1—P1	−174.3 (2)	C27—N6—C28—C29	40.8 (6)
O4—S1—C1—C2	−67.9 (11)	C27—N6—C28—C33	−141.4 (5)
O4—S1—C1—C6	116.9 (9)	C28—C29—C30—C31	−0.3 (11)
O4—S1—C1A—C6A	117.5 (13)	C29—C28—C33—C32	1.6 (8)
O4—S1—C1A—C2A	−63.3 (12)	C29—C30—C31—C32	1.4 (11)
O5—S1—N1—P1	−46.1 (3)	C30—C31—C32—C33	−1.0 (10)
O5—S1—C1—C2	170.6 (8)	C31—C32—C33—C28	−0.5 (9)
O5—S1—C1—C6	−4.6 (12)	C33—C28—C29—C30	−1.1 (8)
O5—S1—C1A—C6A	−5.3 (17)	C34—N7—C35—C36	−58.5 (6)
O5—S1—C1A—C2A	173.9 (9)	C34—N7—C35—C40	121.8 (5)
O6—S2—O7—La1	111.0 (3)	C35—C36—C37—C38	0.3 (10)
O6—S2—N2—P2	−123.1 (4)	C36—C35—C40—C39	−0.2 (7)
O6—S2—C21—C22	−142.0 (6)	C36—C37—C38—C39	0.6 (10)
O6—S2—C21—C26	38.7 (7)	C37—C38—C39—C40	−1.4 (10)
O6—S2—C21A—C26A	36.0 (11)	C38—C39—C40—C35	1.2 (8)
O6—S2—C21A—C22A	−143.9 (7)	C40—C35—C36—C37	−0.5 (8)
O7—S2—N2—P2	6.5 (5)	C41—S3—O9—La1	66.7 (2)
O7—S2—C21—C22	99.8 (7)	C41—S3—N3—P3	−100.0 (2)
O7—S2—C21—C26	−79.6 (7)	C41—C46—C45—C44	0.0
O7—S2—C21A—C26A	−86.3 (10)	C46—C41—C42—C43	0.0
O7—S2—C21A—C22A	93.9 (8)	C46—C45—C44—C43	0.0
O8—S3—O9—La1	−177.3 (2)	C45—C44—C43—C42	0.0
O8—S3—N3—P3	143.7 (2)	C44—C43—C42—C41	0.0
O8—S3—C41—C46	−42.6 (2)	C42—C41—C46—C45	0.0
O8—S3—C41—C42	139.03 (19)	C47—N8—C48—C49	−41.5 (6)
O9—S3—N3—P3	15.2 (3)	C47—N8—C48—C53	137.7 (5)
O9—S3—C41—C46	77.96 (18)	C48—C49—C50—C51	−1.3 (9)
O9—S3—C41—C42	−100.41 (19)	C49—C48—C53—C52	−2.0 (7)
N1—S1—O5—La1	14.1 (3)	C49—C50—C51—C52	0.7 (9)
N1—S1—C1—C2	50.8 (12)	C50—C51—C52—C53	−0.8 (9)
N1—S1—C1—C6	−124.3 (9)	C51—C52—C53—C48	1.5 (8)
N1—S1—C1A—C6A	−125.4 (13)	C53—C48—C49—C50	1.9 (7)
N1—S1—C1A—C2A	53.8 (12)	C54—N9—C55—C56	−39.3 (5)
N1—P1—O1—La1	16.2 (3)	C54—N9—C55—C60	138.9 (4)
N1—P1—N4—C7	2.5 (4)	C55—C56—C57—C58	0.2 (9)
N1—P1—N4—C8	−177.8 (3)	C56—C55—C60—C59	−1.7 (7)
N1—P1—N5—C14	112.8 (3)	C56—C57—C58—C59	−1.4 (10)
N1—P1—N5—C15	−63.0 (7)	C57—C58—C59—C60	1.0 (10)
N1—P1—N5—C15A	−68.6 (9)	C58—C59—C60—C55	0.6 (8)
N2—S2—O7—La1	−18.5 (4)	C60—C55—C56—C57	1.3 (7)
N2—S2—C21—C22	−23.1 (8)	C61—N10—C65—C64	0.2 (5)
N2—S2—C21—C26	157.5 (5)	C61—N10—C65—C66	−178.3 (3)
N2—S2—C21A—C26A	154.9 (8)	C61—C62—C63—C64	0.5 (7)
N2—S2—C21A—C22A	−24.9 (8)	C62—C63—C64—C65	0.6 (7)
N2—P2—O2—La1	14.7 (3)	C62—C63—C64—C67	−179.4 (4)
N2—P2—N6—C27	−6.2 (4)	C63—C64—C65—N10	−1.0 (6)
N2—P2—N6—C28	160.0 (3)	C63—C64—C65—C66	177.6 (4)
N2—P2—N7—C34	−56.6 (4)	C63—C64—C67—C68	−179.4 (4)
N2—P2—N7—C35	131.4 (4)	C64—C65—C66—N11	−176.5 (3)
N3—S3—O9—La1	−49.8 (3)	C64—C65—C66—C69	1.5 (5)
N3—S3—C41—C46	−162.13 (17)	C64—C67—C68—C69	2.1 (7)
N3—S3—C41—C42	19.5 (2)	C65—N10—C61—C62	0.9 (6)
N3—P3—O3—La1	−51.9 (2)	C65—C64—C67—C68	0.7 (7)
N3—P3—N8—C47	13.8 (4)	C65—C66—C69—C68	1.1 (5)
N3—P3—N8—C48	−164.2 (3)	C65—C66—C69—C70	−177.1 (4)
N3—P3—N9—C54	52.0 (3)	C66—N11—C72—C71	0.1 (6)
N3—P3—N9—C55	−97.3 (3)	C66—C69—C70—C71	0.1 (6)
N4—P1—O1—La1	−102.5 (2)	C67—C64—C65—N10	179.0 (4)
N4—P1—N1—S1	158.3 (2)	C67—C64—C65—C66	−2.5 (6)
N4—P1—N5—C14	−134.8 (3)	C67—C68—C69—C66	−3.0 (7)
N4—P1—N5—C15	49.4 (7)	C67—C68—C69—C70	175.1 (4)
N4—P1—N5—C15A	43.8 (9)	C68—C69—C70—C71	−178.1 (4)
N4—C8—C9—C10	178.4 (5)	C69—C70—C71—C72	−0.9 (7)
N4—C8—C13—C12	−177.4 (5)	C70—C71—C72—N11	0.8 (7)
N5—P1—O1—La1	141.5 (2)	C72—N11—C66—C65	177.0 (3)
N5—P1—N1—S1	−87.6 (3)	C72—N11—C66—C69	−1.0 (5)
N5—P1—N4—C7	−116.1 (3)	C21A—S2—O7—La1	−129.7 (7)
N5—P1—N4—C8	63.6 (3)	C21A—S2—N2—P2	121.1 (7)
N5—C15—C20—C19	177.6 (10)	C21A—C26A—C25A—C24A	−0.1 (3)
N5—C15—C16—C17	−177.6 (10)	C26A—C21A—C22A—C23A	−0.1 (6)
N5—C15A—C20A—C19A	−179.7 (14)	C26A—C25A—C24A—C23A	0.2 (7)
N5—C15A—C16A—C17A	179.8 (12)	C25A—C24A—C23A—C22A	−0.3 (9)
N6—P2—O2—La1	−106.2 (3)	C24A—C23A—C22A—C21A	0.3 (8)
N6—P2—N2—S2	120.4 (4)	C22A—C21A—C26A—C25A	0.0 (3)
N6—P2—N7—C34	54.7 (4)	C15A—C20A—C19A—C18A	0.0
N6—P2—N7—C35	−117.3 (4)	C20A—C15A—C16A—C17A	0.0
N6—C28—C29—C30	176.7 (5)	C20A—C19A—C18A—C17A	0.0
N6—C28—C33—C32	−176.3 (5)	C19A—C18A—C17A—C16A	0.0
N7—P2—O2—La1	137.1 (2)	C18A—C17A—C16A—C15A	0.0
N7—P2—N2—S2	−127.5 (4)	C16A—C15A—C20A—C19A	0.0
N7—P2—N6—C27	−120.4 (4)	C1A—S1—O5—La1	−100.8 (10)
N7—P2—N6—C28	45.8 (3)	C1A—S1—N1—P1	70.5 (8)
N7—C35—C36—C37	179.8 (5)	C1A—C6A—C5A—C4A	1.2 (16)
N7—C35—C40—C39	179.5 (4)	C6A—C1A—C2A—C3A	2.2 (15)
N8—P3—O3—La1	70.8 (2)	C6A—C5A—C4A—C3A	3.0 (18)
N8—P3—N3—S3	−99.3 (3)	C5A—C4A—C3A—C2A	−4.5 (18)
N8—P3—N9—C54	−59.5 (3)	C4A—C3A—C2A—C1A	1.8 (16)
N8—P3—N9—C55	151.3 (3)	C2A—C1A—C6A—C5A	−3.8 (15)

### Hydrogen-bond geometry (Å, º)

**Table:**

*D*—H···*A*	*D*—H	H···*A*	*D*···*A*	*D*—H···*A*
C70—H70···O4^i^	0.93	2.67	3.444 (6)	139
C56—H56···O4^ii^	0.93	2.71	3.448 (5)	136

Symmetry codes: (i) *x*+1, *y*, *z*; (ii) *x*+1/2, −*y*+1/2, *z*+1/2.

## References

[bb1] Adams, L. A., Cox, R. J., Gibson, J. S., Mayo-Martín, M. B., Walter, M. & Whittingham, W. (2002). *Chem. Commun.* pp. 2004–2005. 10.1039/b206199f 12357751

[bb2] Agilent (2016). *CrysAlis PRO*. Agilent Technologies Inc., Yarnton, England.

[bb3] Amirkhanov, V., Janczak, C., Macalik, L., Hanuza, J. & Legendziewicz, J. (1995). *J. Appl. Spectrosc.* **62**, 613–624.

[bb4] Amirkhanov, O. V., Moroz, O. V., Znovjyak, K. O., Sliva, T. Y., Penkova, L. V., Yushchenko, T., Szyrwiel, L., Konovalova, I. S., Dyakonenko, V. V., Shishkin, O. V. & Amirkhanov, V. M. (2014). *Eur. J. Inorg. Chem.* **2014**, 3720–3730.

[bb5] Beloso, I., Castro, J., García-Vázquez, J. A., Pérez-Lourido, P., Romero, J. & Sousa, A. (2003). *Polyhedron*, **22**, 1099–1111.

[bb6] Dolomanov, O. V., Bourhis, L. J., Gildea, R. J., Howard, J. A. K. & Puschmann, H. (2009). *J. Appl. Cryst.* **42**, 339–341.

[bb7] Gawryszewska, P., Moroz, O. V., Trush, V. A., Kulesza, D. & Amirkhanov, V. M. (2011). *J. Photochem. Photobiol. Chem.* **217**, 1–9.

[bb8] Gholivand, K., Molaei, F., Oroujzadeh, N., Mobasseri, R. & Naderi-Manesh, H. (2014). *Inorg. Chim. Acta*, **423**, 107–116.

[bb9] Gholivand, K., Oroujzadeh, N. & Rajabi, M. (2012). *J. Iran. Chem. Soc.* **9**, 865–876.

[bb10] Grimes, K. D., Lu, Y.-J., Zhang, Y.-M., Luna, V. A., Hurdle, J. G., Carson, E. I., Qi, J., Sucheta Kudrimoti, S., Rock, C. O. & Lee, R. E. (2008). *ChemMedChem*, **12**, 1936–1945. 10.1002/cmdc.200 PMC2722063 19016283

[bb11] Groom, C. R., Bruno, I. J., Lightfoot, M. P. & Ward, S. C. (2016). *Acta Cryst.* B**72**, 171–179. 10.1107/S2052520616003954 PMC4822653 27048719

[bb12] Jaroslav, K. & Swerdloff, F. (1985). US Patent 4 517 003.

[bb13] Kariaka, N. S., Trush, V. A., Medviediev, V. V., Sliva, T. Y. & Amirkhanov, V. M. (2013). *Acta Cryst.* E**69**, m143. 10.1107/S1600536813003462 PMC3588435 23476491

[bb14] Kasprzycka, E., Trush, V. A., Amirkhanov, V. M., Jerzykiewicz, L., Malta, O. L., Legendziewicz, J. & Gawryszewska, P. (2016). *Chem. Eur. J.* **22**, 1–14. 10.1002/chem.201603767 27781320

[bb15] Kirsanov, A. & Shevchenko, V. (1954). *Zh. Obshch. Khim.* pp. 1980–1993.

[bb16] Kishino, S. & Saito, S. (1979). US Patent 4 161 524.

[bb17] Kovalchyk, T. V., Kudryavtseva, I. G., Sharykina, N. I. & Arzyaeva, E. A. (1991). *Khim. Farm. Zh.* **6**, 63–64.

[bb18] Kulesza, D., Sobczyk, M., Legendziewicz, J., Moroz, O. & Amirkhanov, V. (2010). *Struct. Chem.* **21**, 425–438.

[bb19] Litsis, O. O., Ovchynnikov, V. A., Scherbatskii, V. P., Nedilko, S. G., Sliva, T. Yu., Dyakonenko, V. V., Shishkin, O. V., Davydov, V. I., Gawryszewska, P. & Amirkhanov, V. M. (2015). *Dalton Trans.* **44**, 15508–15522. 10.1039/c5dt02557e 26239675

[bb20] Litsis, O. O., Ovchynnikov, V. A., Sliva, T. Y., Konovalova, I. S. & Amirkhanov, V. M. (2010). *Acta Cryst.* E**66**, m426–m427. 10.1107/S1600536810009670 PMC2983831 21580523

[bb21] Litsis, O. O., Shatrava, I. O., Amirkhanov, O. V., Ovchynnikov, V. A., Sliva, T. Yu., Shishkina, S. V., Dyakonenko, V. V., Shishkin, O. V. & Amirkhanov, V. M. (2016). *Struct. Chem.* **27**, 341–355.

[bb22] Litsis, O. O., Sliva, T. Y., Amirkhanov, V. M., Kolomzarov, Y. V. & Minakova, I. E. (2017). *Proc. Int. Conf. Adv. Optoelectronics and Lasers, CAOL.*, Article number 7851409, 151-153.

[bb23] Morgalyuk, V. P., Safiulina, A. M., Tananaev, I. G., Goryunov, E. I., Goryunova, I. B., Molchanova, G. N., Baulina, T. V., Nifant’ev, E. E. & Myasoedov, B. F. (2005). *Dokl. Chem.* **403**, 126–128.

[bb24] Moroz, O. V., Shishkina, S. V., Trush, V. A., Sliva, T. Y. & Amirkhanov, V. M. (2007). *Acta Cryst.* E**63**, m3175–m3176.

[bb25] Moroz, O., Trush, V., Znovjyak, K., Konovalova, I., Omelchenko, I., Sliva, T., Shishkin, O. & Amirkhanov, V. (2012). *J. Mol. Struct.* **1017**, 109–114.

[bb26] Oroujzadeh, N., Gholivand, K. & Jamalabadi, N. R. (2017). *Polyhedron*, **122**, 29–38.

[bb27] Porai-Koshits, M. & Aslanov, L. (1972). *Zh. Strukt. Khim.* **13**, 266–276.

[bb28] Rogachev, A., Minacheva, L., Sergienko, V., Malkerova, I., Alikhanyan, A., Stryapan, V. & Kuzmina, N. (2005). *Polyhedron*, **24**, 723–729.

[bb29] Safiulina, A. M., Matveeva, A. G., Lizunov, A. V., Bodrin, G. V., Goryunov, E. I., Grigor’ev, M. S., Semenov, A. A., Brel, V. K. & Nifant’ev, E. E. (2015). *Dokl. Chem.* **460**, 57–60.

[bb30] Shatrava, I., Gubina, K., Ovchynnikov, V., Dyakonenko, V. & Amirkhanov, V. (2016*a*). *Acta Cryst.* E**72**, 1683–1686. 10.1107/S2056989016017035 PMC5137587 27980809

[bb31] Shatrava, I., Ovchynnikov, V., Gubina, K., Shishkina, S., Shishkin, O. & Amirkhanov, V. (2016*b*). *Struct. Chem.* **27**, 1413–1425.

[bb32] Shatrava, I. O., Sliva, T. Y., Ovchynnikov, V. A., Konovalova, I. S. & Amirkhanov, V. M. (2010). *Acta Cryst.* E**66**, m397–m398. 10.1107/S1600536810008214 PMC2983777 21580501

[bb33] Sheldrick, G. M. (2015*a*). *Acta Cryst.* A**71**, 3–8.

[bb34] Sheldrick, G. M. (2015*b*). *Acta Cryst.* C**71**, 3–8.

[bb35] Sokolnicki, J., Legendziewicz, J., Amirkhanov, W., Ovchinnikov, V., Macalik, L. & Hanuza, J. (1999). *Spectrochim. Acta A*, **55**, 349–367.

[bb41] Spek, A. L. (2015). *Acta Cryst* C**71**, 9–18. 10.1107/S2053229614024929 25567569

[bb36] Xu, K. & Angell, C. A. (2000). *Inorg. Chim. Acta*, **298**, 16–23.

[bb37] Yizhak, R. V., Znovjyak, K. O., Ovchynnikov, V. A., Sliva, T. Y., Konovalova, I. S., Medviediev, V. V., Shishkin, O. V. & Amirkhanov, V. M. (2013). *Polyhedron*, **62**, 293–299.

[bb38] Zefirov, N. S., Palyulin, V. A. & Dashevskaya, E. E. (1990). *J. Phys. Org. Chem.* **3**, 147–158.

[bb39] Znovjyak, K. O., Ovchynnikov, V. A., Sliva, T. Y., Shishkina, S. V. & Amirkhanov, V. M. (2009). *Acta Cryst.* E**65**, o2812. 10.1107/S1600536809042391 PMC2971180 21578403

[bb40] Znovjyak, K. O., Seredyuk, M., Kusz, J., Nowak, M., Moroz, O. V., Sliva, T. Yu. & Amirkhanov, V. M. (2015). *J. Mol. Struct.* **1100**, 145–149.

